# Unveiling the Algorithm: The Role of Explainable Artificial Intelligence in Modern Surgery

**DOI:** 10.3390/healthcare13243208

**Published:** 2025-12-08

**Authors:** Sara Lopes, Miguel Mascarenhas, João Fonseca, Maria Gabriela O. Fernandes, Adelino F. Leite-Moreira

**Affiliations:** 1 Portuguese Institute of Oncology of Porto, 4200-072 Porto, Portugal; 2 Faculty of Medicine, University of Porto, 4200-437 Porto, Portugal; 3 Precision Medicine Unit, Department of Gastroenterology, Hospital São João, 4200-437 Porto, Portugal; 4 WGO Training Center, 4200-437 Porto, Portugal; 5 Institute for Research and Innovation in Health—Associate Laboratory (i3s-LA) (IPATIMUP/I3S), 4200-135 Porto, Portugal; 6 Department of Cardiothoracic Surgery, Hospital São João, 4200-437 Porto, Portugal

**Keywords:** artificial intelligence, explainable artificial intelligence, XAI, surgery, bioethics, generative AI, ChatGPT

## Abstract

Artificial Intelligence (AI) is rapidly transforming surgical care by enabling more accurate diagnosis and risk prediction, personalized decision-making, real-time intraoperative support, and postoperative management. Ongoing trends such as multi-task learning, real-time integration, and clinician-centered design suggest AI is maturing into a safe, pragmatic asset in surgical care. Yet, significant challenges, such as the complexity and opacity of many AI models (particularly deep learning), transparency, bias, data sharing, and equitable deployment, must be surpassed to achieve clinical trust, ethical use, and regulatory approval of AI algorithms in healthcare. Explainable Artificial Intelligence (XAI) is an emerging field that plays an important role in bridging the gap between algorithmic power and clinical use as surgery becomes increasingly data-driven. The authors reviewed current applications of XAI in the context of surgery—preoperative risk assessment, surgical planning, intraoperative guidance, and postoperative monitoring—and highlighted the absence of these mechanisms in Generative AI (e.g., ChatGPT). XAI will allow surgeons to interpret, validate, and trust AI tools. XAI applied in surgery is not a luxury: it must be a prerequisite for responsible innovation. Model bias, overfitting, and user interface design are key challenges that need to be overcome and will be explored in this review to achieve the integration of XAI into the surgical field. Unveiling the algorithm is the first step toward a safe, accountable, transparent, and human-centered surgical AI.

## 1. Introduction

While it is entering all aspects of our lives, mainly through Generative Artificial Intelligence (GenAI), AI is revolutionizing healthcare. Indeed, GenAI also has applications in Medicine [Table 1]. In particular, large language models (LLMs) have been a focus of intense research, motivated by the recent introduction of Chat Generative Pre-Trained Transformer (ChatGPT, OpenAI). ChatGPT (OpenAI, San Francisco, CA, USA) has been commercially developed since 2022, as a tool that understands, generates, and interacts with textual inputs, allowing extensive data interpretation, with potential in answering clinical questions, assisting differential diagnostic processes: it can provide patient recommendations and elaborate research questions [1,2]. Table 2 summarizes studies that evaluated LLM tools’ performance for clinical diagnosis [2,3,4,5,6,7,8].

But in surgery, image analysis is of utmost importance, and Deep Learning (DL) models have demonstrated high accuracy in detection and characterization of lesions across distinct imaging methods, in Gastroenterology, using neural architectures adapted for image analysis, mainly convolutional neural network (CNN) models [7]. Recent versions of Generative AI (ChatGPT-4) have opened the possibility of integrating image interpretation with clinical narratives, by the introduction of visual capabilities. However, they lag behind specialized CNN models, typically used in clinical image interpretation, that are optimized for medical image classification and segmentation (Table 3 compares LLMs and CNNs). Mascarenhas et al. [1] highlighted that while the emergence of image-capable LLMs is promising, ChatGPT-4 currently falls short in terms of accuracy and consistency, and lacks explainable mechanisms. These authors investigated ChatGPT-4’s performance in interpreting images in Gastroenterology across five diagnostic modalities. A total of 740 images were assessed using standardized prompts, and ChatGPT-4’s image interpretations were compared with established gold-standard diagnoses. Performance metrics included accuracy, sensitivity, specificity, positive and negative predictive values, and area under the curve (AUC). Accuracy ranged from 50% to 90% in capsule endoscopy (CE), 67% in device-assisted enteroscopy (DAE) broadly, and under 70% in high-resolution anoscopy (HRA). Critically, discriminative performance (AUC) was notably poor in Endoscopic Ultrasound (EUS) and digital single-operator cholangioscopy (DSOC), indicating inadequate reliability [1].

It is important to distinguish between model families (e.g., CNNs, transformers, diffusion models, LLMs), task types, and explanation layers. XAI methods such as Grad-CAM, SHAP, attention rollout, or counterfactuals operate on top of a model and do not depend on whether the underlying model is generative or discriminative. This clean separation clarifies that explainable artificial intelligence (XAI) methods operate independently of model family, avoids conflating GenAI limitations (e.g., hallucination, lack of pixel-level fidelity) with XAI requirements, and the independent question of explanation faithfulness and stability.

Importantly, the maturity of evidence supporting XAI varies substantially across surgical subspecialties. Fields such as radiology, pathology, and ophthalmology, where data are highly structured and outcomes are readily quantifiable, have generated more rigorous validation studies and clearer demonstrations of how XAI can enhance diagnostic confidence and workflow integration. In contrast, subspecialties like emergency general surgery, trauma, and complex oncologic surgery often rely on heterogeneous data sources, non-standardized documentation, and clinically nuanced decision-making, resulting in fewer high-quality studies and limited reproducibility of XAI models. These differences highlight that enthusiasm for XAI is not uniformly matched by methodological robustness, and that the translation of XAI into practice will require subspecialty-specific frameworks for data curation, model evaluation, and reporting standards.

In summary, GenAI’s most promising role today lies in generating synthetic training datasets, improving image quality, or assisting in patient communication. LLMs, like ChatGPT-4, are not XAI and need substantial enhancement before integration into diagnostic workflows [1]. Accuracy, consistency, and explainability will be essential to bridge trust, accountability, and safe integration into surgical and diagnostic workflows.

### 1.1. The Rise of AI in Surgery

**Figure 1:**
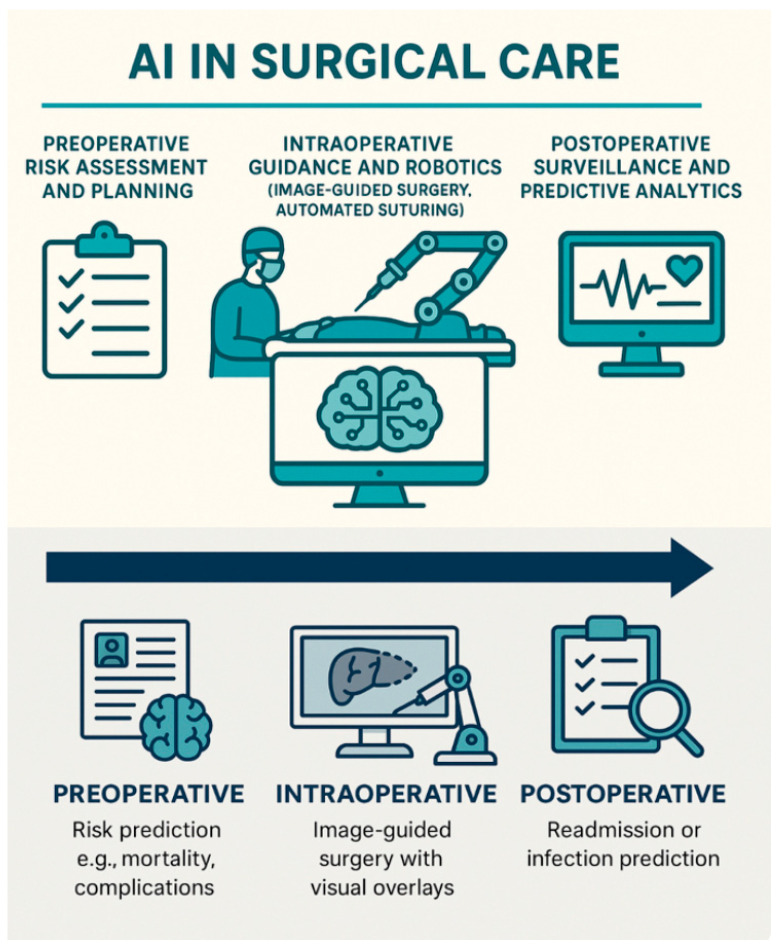
Artificial Intelligence (AI) and Explainable Artificial Intelligence (XAI) in surgical care: preoperative, intraoperative, and postoperative.

AI tools applied in surgery are now extending beyond theoretical models: they are allowing tangible enhancement of efficiency, precision, and outcomes. From the outpatient clinic to the operating theater and postoperative wards, over the past years, AI has emerged as a powerful innovation tool across all phases of surgical care. Applications of AI in surgical care include preoperative risk assessment and planning; intraoperative guidance, and robotics (image-guided surgery, automated suturing); and postoperative surveillance, and predictive analytics [Figure 1].

Preoperative risk assessment and planning is a challenging and game-changing area of advancement in AI, that is continually growing in scope of application, and is already surpassing traditional tools in accuracy and workflow efficiency. AI-driven risk stratification tools are increasingly reshaping traditional workflows, also helping surgeons in personalized optimization strategies [10].

**Figure 2:**
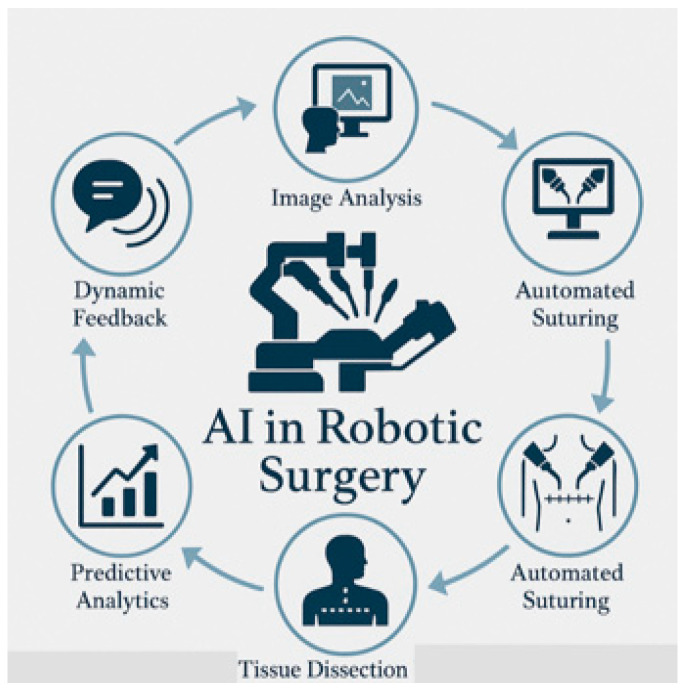
Through AI-powered robotic systems (e.g., Da Vinci Surgical System), AI provides real-time insights during minimally invasive procedures with image analysis, automated suturing, predictive analytics, tissue dissection under supervised control, and dynamic feedback.

Intraoperatively, AI-enhanced and AI-driven Surgical Planning Systems (e.g., Medtronic’s AI-powered navigation) are enabling real-time navigation and decision support [11]. Robotic-assisted surgery (RAS), once primarily focused on mechanical precision, has entered a new phase of intelligence, with emerging platforms capable of semi-autonomous tasks [Figure 2].

**Figure 3:**
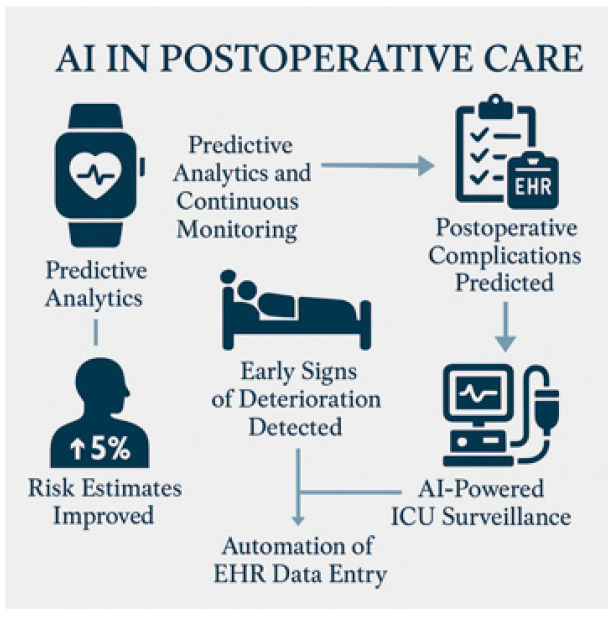
AI in postoperative care. By identifying high-risk patients weeks ahead, clinicians may improve comorbidity control and also provide an early intervention. AI algorithms improved physicians’ risk estimates by up to 5%, enhancing identification of postoperative outcomes. AI helps with ventilator management in ICUs, optimizing respiratory support for critically ill patients. EHR—Electronic Health Records; ICU—Intensive Care Unit.

Postoperative care is also undergoing a transformation through predictive analytics and continuous monitoring [10]. AI algorithms embedded in wearable devices and Electronic Health Records (EHR) systems can detect early signs of deterioration, predict postoperative complications, and support timely interventions [12,13,14] [Figure 3]. AI also assists with AI-powered intensive care unit (ICU) surveillance.

**Figure 4:**
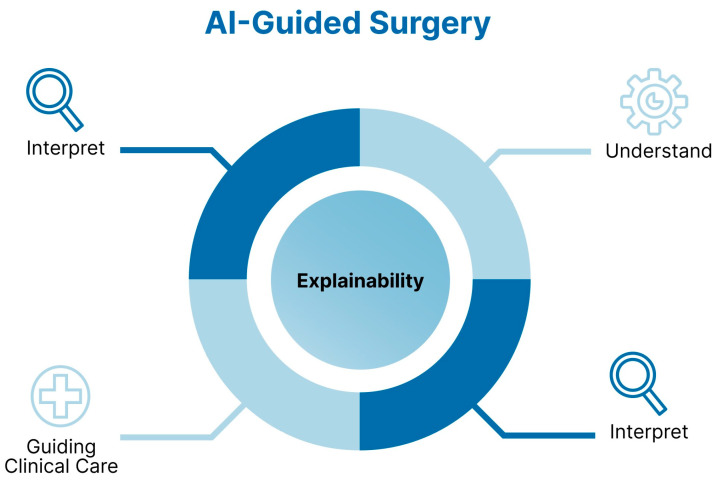
The rise of AI in surgery must be met with an equally strong emphasis on explainability: ensuring that surgical teams understand, interpret, and can critically evaluate the algorithms, guiding clinical care.

Collectively, all these innovations signal a paradigm shift in modern surgery. Yet, as algorithms grow more complex, the opacity of their decision-making processes poses a new challenge: trust. The rise of AI in surgery must be met with an equally strong emphasis on explainability [Figure 4]. However, Explainable AI is not a magic bullet for “trust”; it is one component of a broader agenda for trustworthy, structurally enabled, clinically integrated AI.

### 1.2. Why Explainability Matters in Surgical AI

**Figure 5:**
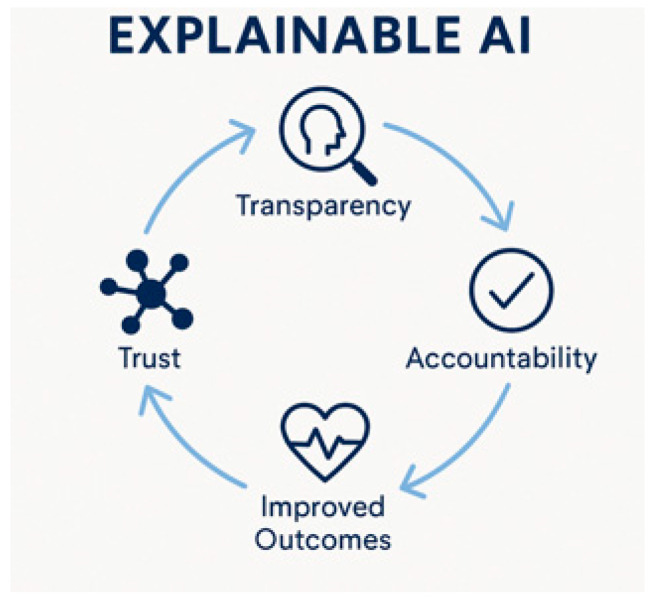
Explainability is a cornerstone for safety, trust, and legal responsibility. XAI techniques translate opaque outputs into actionable insights.

Surgical teams operate in high-stakes, time-sensitive settings, and many machine learning (ML) models work as “black boxes.” Beyond their technical prowess, AI systems in surgery must be thoughtfully integrated into clinical environments to ensure ethics, safety, and trust. Complexity, without explainability and transparency, might produce clinicians’ distrust, or misuse predictions [15] [Figure 5].

Although the explainability and trustworthiness are related, they are distinct targets in clinical AI: explainability is a property of models and interfaces (what, how, and to whom things are explained), and trustworthiness is a property of the entire socio-technical system (including validation, governance, infrastructure, and institutional culture). Keeping them separate actually helps make sense of why many promising tools fail to cross the translational gap [16].

**Figure 6:**
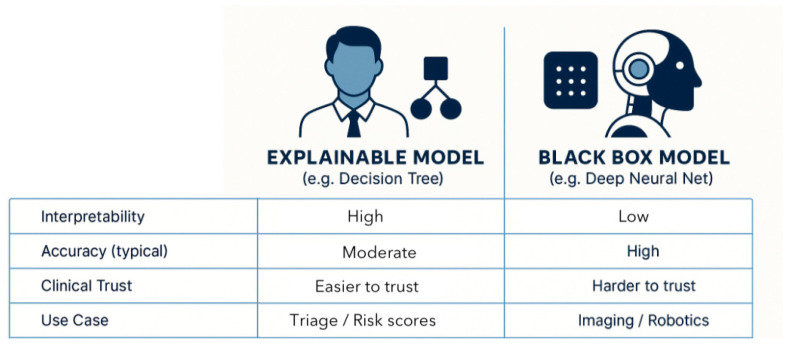
Explainable versus ‘Black box’ models: a side-by-side comparison. Explainable/interpretable models (e.g., decision trees, linear/logistic regression) are white-box models. Black-box models, especially deep neural networks (DNNs), offer superior performance on complex tasks such as image-based intraoperative guidance but lack transparency without additional methods. Simpler, interpretable models offer transparency but may underperform at capturing intricate patterns.

Shapley additive explanations (SHAP), local interpretable model-agnostic explanations (LIME), saliency maps, attention maps, and natural-language summaries, are examples of XAI techniques that support AI integration in surgery, by unveiling the reasoning behind algorithmic recommendations: XAI enables human oversight, surpassing ‘the black box’ problem, which will foster clinical trust and usability, supporting educational feedback, and adoption in clinical workflows [17] [Figure 6].

XAI is an important, but incomplete strategy: it supports informed scrutiny and can underpin warranted trust, but only within institutions that have the regulatory, infrastructural, and cultural capacity to act on what explanations reveal.

### 1.3. Aim of the Review

Integration of AI into surgical practice will dramatically improve patient care, offering newfound opportunities to enhance precision, efficiency, and outcomes. However, surgeons should be aware of both the strengths and limitations of AI-based tools.

ML algorithms have shown surprisingly satisfactory predictive performances in many studies, but these tools are not immune to small sample sizes, requiring ‘big data’ to ensure predictions [10]. ML-based tools can analyze large, high-dimensional, and unstructured data; however, this ability can come at the expense of complexity, leading to the ‘black box’ problem (lack of transparency and interpretability) [10].

We can say that explainability could be the path to promoting equitable care. XAI contributes to reproducibility, debugging, and safety: explainability in AI underpins core medical ethics (e.g., autonomy, beneficence, non-maleficence, and justice), by enabling clinicians and patients to understand and critically evaluate model outputs (AI-driven decisions), for accuracy and fairness [18]. Transparent explanations will allow validity, meaningful monitoring, and identification of vulnerabilities (e.g., biased predictions). They will also support regulatory compliance, data governance, and cybersecurity (through algorithms that are not only powerful, but also transparent and auditable) [18,19]. However, there are important obstacles from explainability to implementation. The main obstacles to AI clinical adoption are often structural, not purely algorithmic. Karamitros et al.’s discussion of AI in Plastic Surgery is a good example: these authors argue that the gap between abundant proofs-of-concept and sparse real-world deployment reflects an organizational readiness deficit rather than a modeling deficit [16]. Several layers of barriers are highlighted in this paper, and can be generalized beyond Plastic Surgery: institutional/organizational, regulatory and governance, and infrastructural and ecosystem barriers.

An infrastructure that allows models to be trained, evaluated, explained, and monitored in environments that resemble real practice, foments trustworthiness; and without it, explanations risk becoming static, decoupled from how the system behaves as practice patterns, patient populations, or upstream data sources shift. There are several key obstacles as data quality and representativeness (biased, incomplete, or poorly labeled datasets cannot be ‘explained’ into trustworthiness); interoperability and integration (lack of robust EHR integration, standardized data formats, and logging infrastructure makes it difficult to surface explanations at the point of care, or to audit model performance across populations and sites); and monitoring and feedback loops (a trustworthy system requires continuous surveillance for performance drift, bias, and failure modes, which in turn needs data pipelines, dashboards, and escalation pathways).

The authors aim to explore the role of AI in surgery, emphasizing the ethical, clinical, and technical role of explainability in current applications and future trajectories, as well as in both ethical and legal challenges. Key applications and future directions of XAI in surgery are summarized in Table 4 and Table 5 [17,18,19,20,21]. In surgery, surgeons must have the power of critical appraisal over AI recommendations, which is essential for AI implementation (especially when using LLMs).

## 2. Methods

This article is a systematic review aiming to synthesize the role of XAI in modern surgical practice. The authors followed transparent selection procedures, conducting the research across databases such as PubMed, Scopus, Web of Science, and arXiv, within the time window of January 2000 and September 2025. ‘Explainable artificial intelligence’, ‘XAI’, ‘surgery’, ‘image-guided’, ‘bioethics’, ‘robotic surgery’, ‘intraoperative’, ‘interpretability’, ‘trustworthiness’, ‘accountability’, ‘deep learning’, ‘LLMs’, ‘GenAI’, ‘Grad-CAM’, ‘SHAP’ and ‘LIME’, were the keywords used.

The authors included studies involving surgical tasks (diagnostic, planning, intraop, postop), the use of an AI model with an explicit interpretability or XAI component, and human or experimental surgical contexts. Purely technical computer vision papers with no surgical relevance, and non-original content (opinions, editorials), were excluded.

We applied a light-weight framework incorporating representativeness, external validation, availability of ground truth, clarity of XAI reporting, and human-factors evaluation (adapted from AMSTAR). Figures from published articles were used with license and approval.

## 3. Foundations of Explainable AI in Surgical Contexts

### 3.1. Definitions and Principles

**Figure 7:**
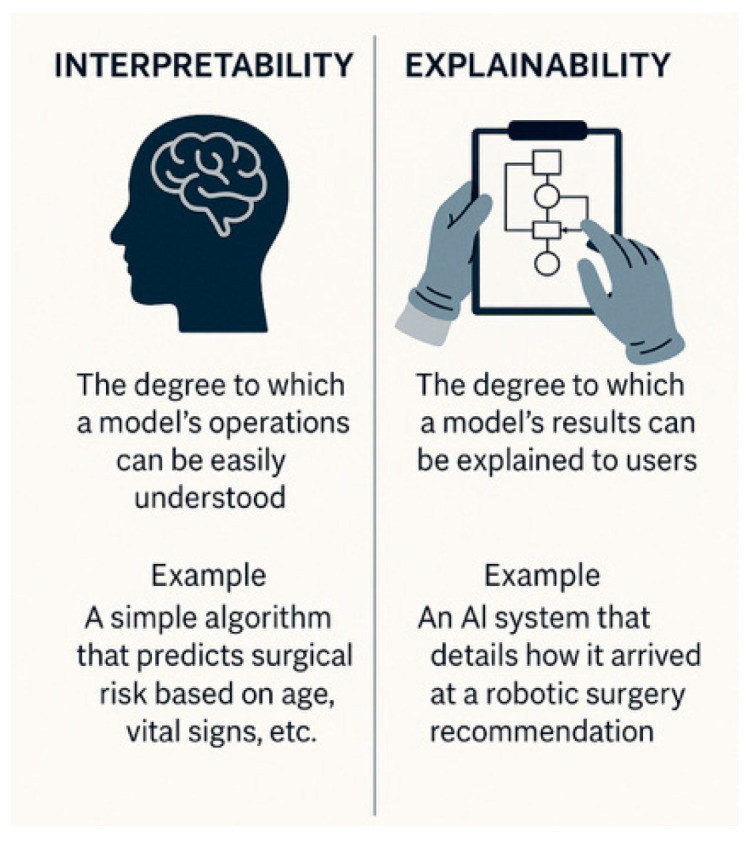
Interpretability—how intuitively a human can understand a model. Interpretability is inherent clarity, while explainability refers to the reasoning behind ‘black-box models’.

When applying AI in surgical contexts, it is important to distinguish interpretability and explainability [Figure 7]. Interpretability refers to the inner logic and how inputs map to outputs. However, by contrast, explainability entails providing transparent reasons for specific decisions, often through post hoc analysis of opaque models [22]. XAI can be defined as the properties and techniques that make a model’s behavior intelligible to human stakeholders. The focus is on how a prediction was reached, in a way that clinicians, patients, and regulators can meaningfully understand and scrutinize. Explainability is about understanding outputs, which can be a contributor to warranted trust (by exposing model logic, surfacing biases, and supporting contestability). A perfectly transparent, but poorly validated, biased, or easily gamed model is explainable, but not trustworthy. Conversely, some high-stakes tools may be trustworthy by virtue of rigorous validation, monitoring, and institutional safeguards, even if explanations are relatively coarse-grained. Trustworthiness is about justified reliance: a grounded belief that the system will behave appropriately under uncertainty and vulnerability (for both patients and clinicians), supported by evidence from design, validation, governance, and oversight: it is a broader socio-technical property of an AI system in context—it concerns whether the system is safe, reliable, fair, robust, privacy-respecting, and appropriately governed, and whether these qualities are demonstrated and verifiable in real clinical settings [16]. FUTURE-AI is a framework that treats trustworthiness as a bundle of requirements spanning data quality, robustness, usability, fairness, transparency, and external validation.

**Figure 8:**
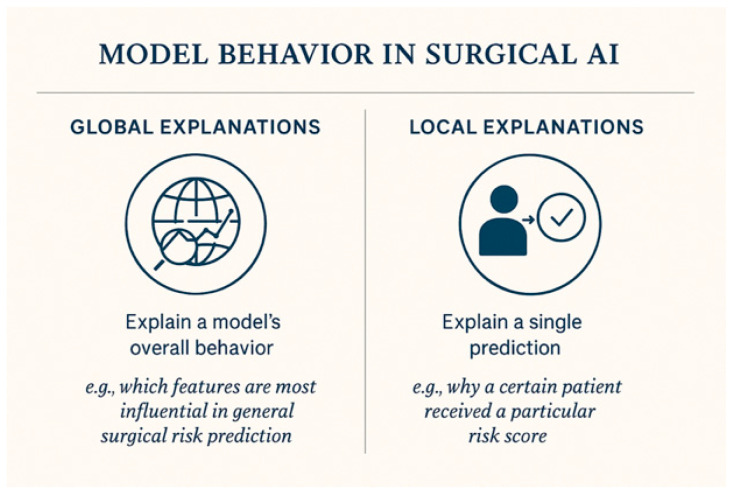
Global and local explanations. Global explanations aim to elucidate a model’s overall behavior (e.g., which features are most influential in general surgical risk prediction, or how model outcomes change with inputs across the dataset). Local views support point-of-care decisions and patient-specific counseling.

In model behavior, both global and local explanations are critical in surgical AI [Figure 8]. Global explanations aim to elucidate a model’s overall behavior, which, in other words, means that global views establish model validity [23,24]. Highlighting contributing factors (e.g., age, comorbidities, lab values), local explanations focus on a single prediction to explain why a certain patient received a particular risk score [22,23,24].

**Figure 9:**
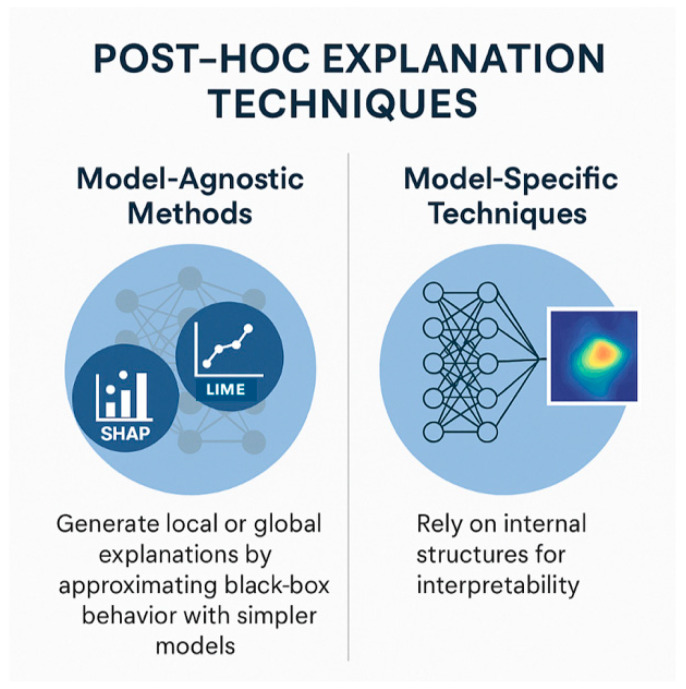
Post hoc explanation techniques. Model-agnostic methods generate local or global explanations by approximating black-box behavior with simpler models, or even assigning feature attribution scores. Model-specific techniques rely on internal structures for interpretability.

Post hoc explanation techniques are used after a model has been trained to help us understand how and why it makes decisions (especially when dealing with complex or opaque models like DNNs). They can be model-agnostic methods (e.g., LIME and SHAP) or model-specific techniques (e.g., salience maps, Grad-CAM in DL, rule extraction for decision trees) [Figure 9] [21,22].

There are explainable and non-explainable models [Figure 6]. Inherently explainable/interpretable models rely on decision paths or coefficients that directly reveal how features influence outcomes [20,21]. Black-box systems excel in accuracy, yet demand XAI to justify outputs. In surgical contexts, XAI is essential to reconcile tension between transparency, accuracy, trust, safety, and compliance [17,20,23].

### 3.2. Key XAI Techniques Used in Surgery

**Figure 10:**
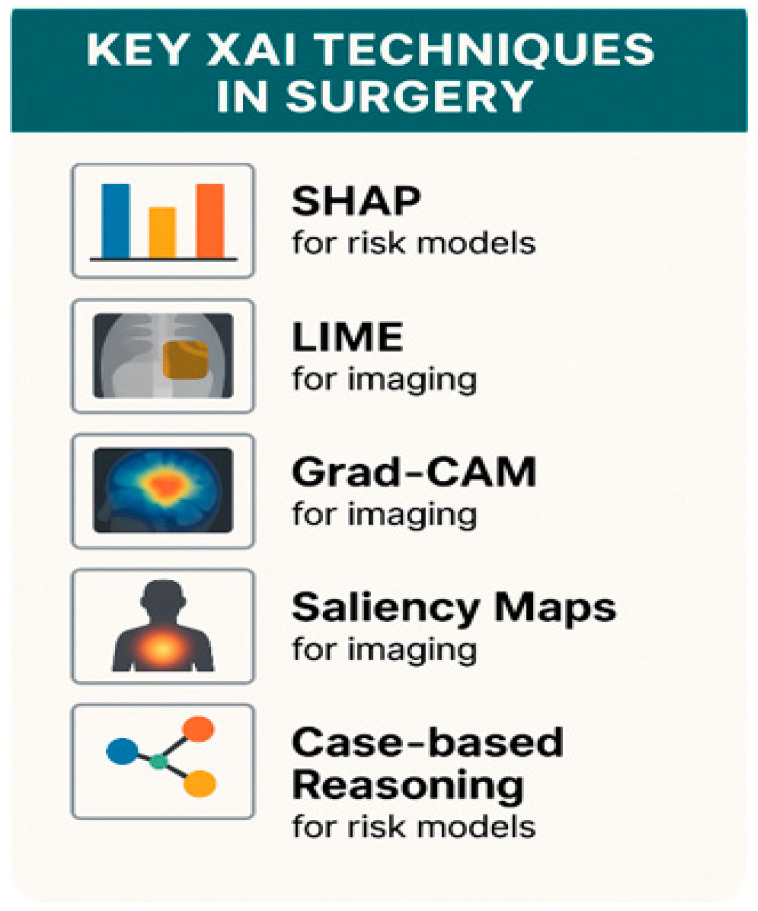
Summary of XAI techniques in surgery.

In surgery, XAI techniques allow feature attribution methods, image visualization techniques, case-based and prototype reasoning, and visual explainability (in robotic and laparoscopic surgeries) [Table 6; Figure 10].

SHAP and LIME are examples of model-agnostic methods (post hoc explanation techniques), which means they are feature explanations. SHAP quantifies each feature’s contribution to a specific decision using a game-theoretic approach, giving clinicians insight into why a risk score was assigned (e.g., whether age or renal function drove the result) [25]. LIME builds simplified local models to explain complex predictions, spotlighting crucial variables (e.g., lab values, comorbidities in preoperative risk models) [25,26]. In high-stakes decisions, as in surgery, LIME’s results can be unstable depending on input perturbations; hence, cautious interpretation is of utmost importance.

**Figure 11:**
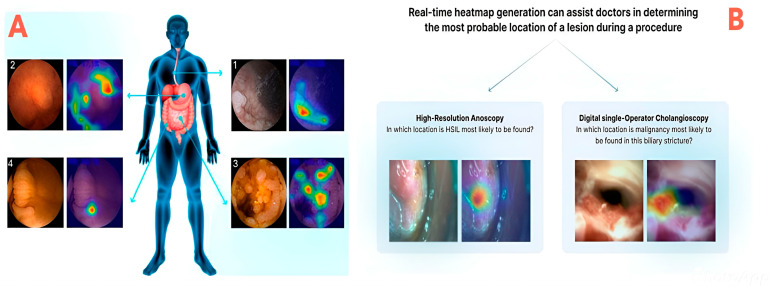
(**A**) Examples of generated heatmaps for different types of lesions in different locations in Capsule Endoscopy. Each prediction is associated with a degree of certainty expressed as a percentage, while the generated heatmap identifies the area responsible for the prediction. The lesions are numbered as follows: 1—P1U-P1 (ulcer lesion by Saurin classification); 2—P1PE (erosion by Saurin Classification); 3—PV (vascular lesion); 4—PP/REST (pleomorphic lesion). (**B**) Real-time heatmap generation for lesion location and biopsy guidance in high-resolution anoscopy and digital single-operator cholangioscopy. Images and legends from [29].

Grad-CAM is a type of image visualization XAI technique (model-specific technique) that is widely adopted in image-based surgical support systems (e.g., tissue characterization or tumor segmentation). It overlays heat maps on medical images (computed tomography—CT, magnetic resonance imaging—MRI, and ultrasound, among others) to indicate which regions influenced the model [24,27,28]. Visual explainability in robotic and laparoscopic systems is achieved through the application of Grad-CAM or salience overlays. Saliency maps (another model-specific technique) are usually combined with Grad-CAM, SmoothGrad, Score-CAM, or Guided-Grad-CAM to improve clarity and reliability, because they highlight pixel-level importance via back propagation. However, they can be noisy. AI-enhanced robotic platforms increasingly integrate explainability by overlaying segmentation maps and attention heatmaps directly on laparoscopic video feeds: this allows for real-time visual integration, which enables surgeons to understand why instruments target specific anatomy (e.g., vessels, tumors). In this way, trust and safety in image-guided interventions can be enhanced [Figure 11].

**Figure 12:**
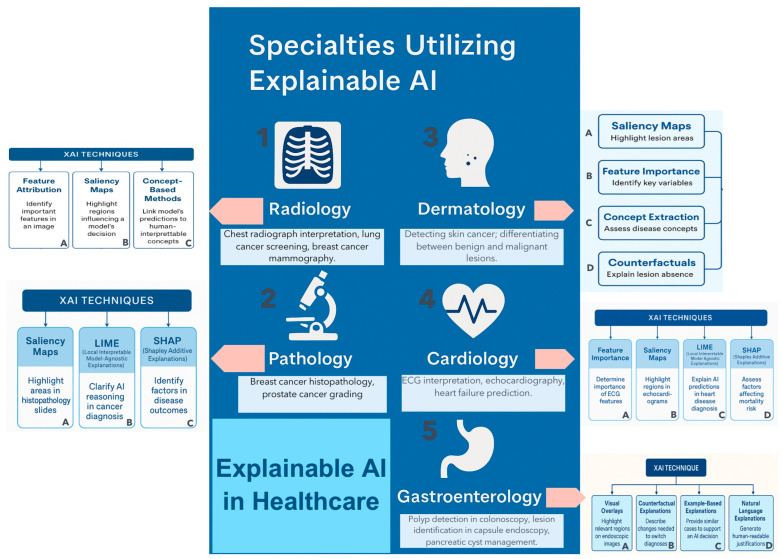
Current landscape of XAI in healthcare. **Radiology**: 1A—(SHAP, LIME)—Identify which image features (density, texture, anatomical area) most contributed to diagnostic predictions; 1B—Highlight regions in chest CTs, MRIs, and mammograms that influence AI-based diagnosis (e.g., tumor or lesion localization); 1C—Link radiologic findings to interpretable clinical features (e.g., “nodule size,” “calcification”). **Pathology** 2A—Visual overlays on histopathology slides, showing regions most responsible for AI classification (e.g., tumor vs. normal tissue); 2B, 2C—Explain cell morphology or tissue features influencing cancer subtype classification. **Dermatology** 3A—Highlight lesion areas that drive classification of melanoma vs. benign nevi. 3B—Identify critical variables (color, asymmetry, border irregularity); 3C—Map predictions to dermatology concepts such as “pigmentation pattern”; 3D—Show what changes (e.g., border smoothness) would alter classification outcome. **Cardiology** 4A—Determines which ECG or echocardiographic features (e.g., QT interval, ventricular dimensions) drive AI predictions; 4B—Localize critical regions in echocardiograms that influence diagnostic outcomes; 4C,D—Provide patient-level explanations for AI-predicted risk of arrhythmia or heart failure. **Gastroenterology** 5A—(Grad-CAM, saliency) Highlight relevant regions on endoscopic or capsule endoscopy images for lesion detection; 5B—Illustrate what minimal changes in image features would switch diagnosis (e.g., from benign polyp to neoplastic lesion); 5C—Provides similar cases to support the AI decision; 5D—Generate human-readable justifications to accompany image analysis.

Case-based and prototype reasoning are methods that surface similar historical cases or image prototypes, which allow surgeons to compare current patients with registered precedents. This ensures that decisions rest on recognized clinical analogies. XAI techniques are beginning to be applied in several areas of Medicine: Radiology [26], Pathology [30], Cardiology [31], Dermatology [32], and with special emphasis in Gastroenterology [29] [Figure 12].

### 3.3. Unique Challenges in the Surgical Domain

Institutional barriers are of utmost importance (and mainly modifiable) [16]. Clinicians may struggle to interpret even good explanations, lack time to engage with model development, or feel sidelined by data-science teams. Early-career ‘AI Champions’ in departments often lack senior sponsorship, protected time, or formal roles to allow linkage of clinical and technical work; and misaligned incentives and underevaluation of data assets (funding, promotion criteria, publication incentives) tend to reward novel models, not robust deployment, external validation, or maintenance.

A constellation of data streams informs modern surgeries: preoperative data (EHRs, lab values, comorbidities); intraoperative imaging (laparoscopic video, fluorescence, ultrasound); real-time sensors (hemodynamics, instrument kinematics); and postoperative metrics (drains, early biomarkers). In this way, integration of multimodal time-synchronized data (video, imaging, EHRs, sensor streams) is a unique challenge of AI in surgery, because existing XAI techniques are often designed for univocal inputs. Only a few frameworks can explain decisions that arise from a complex fusion of structured and unstructured sources, limiting clinicians’ ability to trust the logic behind outputs [33,34].

Dynamic, high-risk, and data-dense surgical environments present unique challenges for AI implementation and explainability. Surgery demands AI systems that are not only accurate but also interpretable, responsive, and seamlessly integrated into clinical workflow: surgeons need a clear answer. Surgery is all about real-time decision-making requirements: surgical decisions often unfold in seconds, unlike static predictions in radiology or pathology. Identifying a critical vessel, deciding the extent of resection, and responding to sudden bleeding are examples of intraoperative scenarios that require AI systems capable of processing and explaining decisions in real time. XAI methods in surgery pretend to deliver low-latency, high-clarity outputs that do not interrupt or slow down the surgical process. However, explanations must be intuitive, concise, and clinically actionable. Visual aids (e.g., heatmaps, overlay annotations) and analogies to past cases are more effective in this context than mathematical models or textual descriptions [35]. However, many current XAI techniques, such as SHAP, are computationally intensive and not optimized for real-time use, highlighting a technical gap between research and bedside application [28,36]. Due to the interdisciplinary nature of surgical teams (e.g., anesthesiologists, residents, nurses), AI outputs must be understandable to all different cognitive profiles and expertise levels [21].

Another big challenge is the lack of standardized validation: a systematic review found that only 45% of surgical AI models present high-quality validation, and only 14% share datasets publicly [15]. Models trained on hospital-specific populations (e.g., hip fracture patients in Taiwan) may not transfer well elsewhere (Data Bias and Generalizability) [12,15].

## 4. Ethical and Legal Imperatives for Explainable AI in Surgery

**Figure 13:**
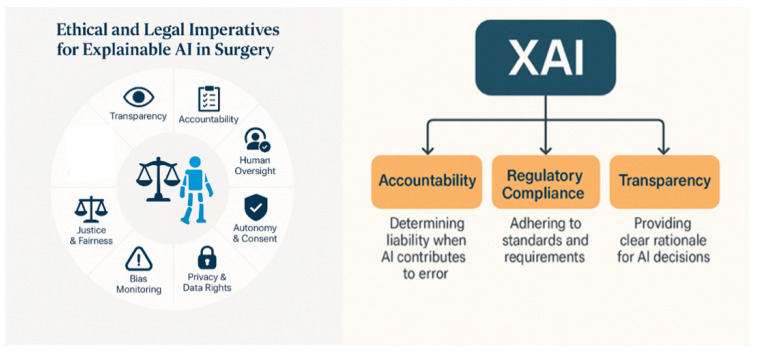
Ethical considerations of XAI in surgery.

Core ethical dimensions of XAI in surgery are summarized in [Figure 13 and Table 7]. XAI systems are designed to make their decision-making process interpretable and understandable: surgeons must understand and evaluate AI-driven recommendations before acting. But even when technical explainability is strong, regulatory and liability concerns remain major brakes on AI clinical deployment. Karamitros et al. emphasize that institutional readiness must include governance structures capable of addressing data governance, reimbursement, and medico-legal questions, and not just the adoption of new algorithms: trustworthiness in medicine is tightly coupled to regulatory clarity and governance, dimensions that sit largely outside the technical scope of XAI methods [16]. Many regulatory regimens still assume relatively static devices, whereas clinical AI models may drift as data and practice evolve. Regardless of how well the model is explained, if clinicians override an AI recommendation that is later judged ‘correct’, or follow a recommendation that turns out harmful, this ambiguity can encourage conservative non-use (Liability and Accountability). Regulators and ethics bodies increasingly call for ‘transparent’, ‘explainable’ or ‘interpretable’ systems, but provide limited concrete criteria for sufficiency, or standardized evaluation metrics for explanations [16].

If there is no shared conceptual language to interpret explanations, nor workflow structures to act on them, the potential of XAI is weakened [16]. Immature human-capital pipelines make it difficult to maintain and iteratively improve deployed systems, undermining long-term trustworthiness, even when initial explainability and performance look promising. High-quality labeling, curation, and documentation of data (central to both trustworthy and explainable AI) are often treated as cost centers rather than core infrastructure. Even highly explainable models may never move beyond pilot projects, or they are deployed without the organizational support needed for ongoing monitoring, calibration, and recalibration of trust.

### 4.1. Accountability and Responsibility

**Figure 14:**
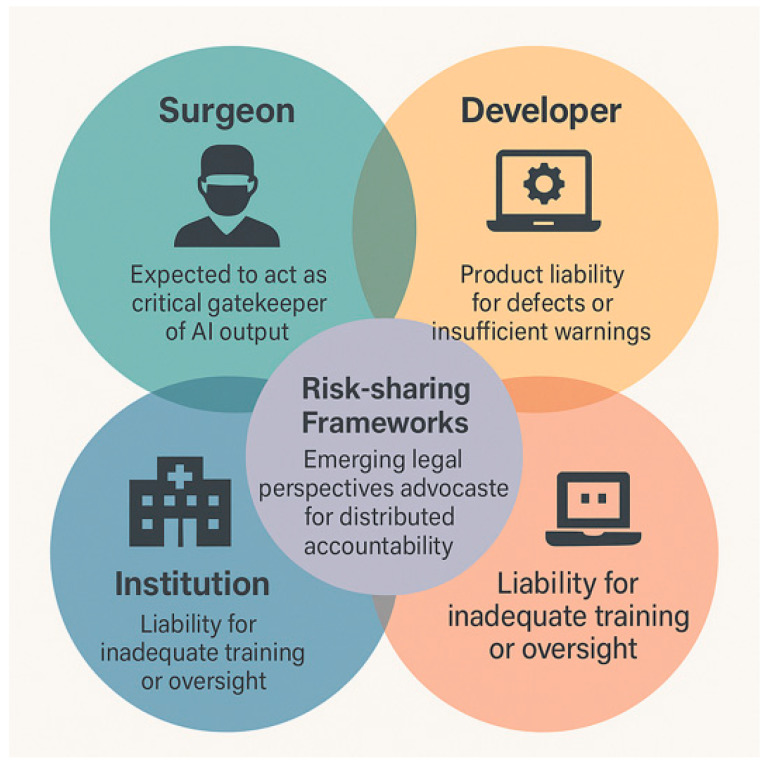
Accountability in XAI surgery: Surgeon—is expected to be the final decision-maker, capable of overriding algorithmic suggestions, if clinically inappropriate; Developer—may be responsible for product liability or failure to warn; Institutions—could face liability for inadequate training, lack of oversight, or failure to properly validate AI models within their specific context; Risk-sharing frameworks—shared responsibility models.

Accountability is one of the ethical and legal imperatives of XAI in surgery. Determining liability when AI contributes to surgical error is a pressing challenge at the intersection of ethics, medicine, and law. In many legal systems, the surgeon is expected to bear ultimate responsibility for AI use during clinical care, even if the system is Food and Drug Administration (FDA)- or European Certified (CE)-approved [37,38,39]. The Developer may be held accountable under product liability laws if harm results from software defects, misrepresentations, or a lack of sufficient warnings about system limitations, and this is particularly relevant for autonomous systems or black-box models with limited clinician oversight [40,41]. Hospital protocols and governance structures are crucial in defining how AI integrates safely into surgical workflows [21]. Emerging legal perspectives advocate for distributed accountability across the clinical–technical–institutional triad. Risk-sharing frameworks may become essential as AI systems increase in autonomy and influence on surgical outcomes [42,43] [Figure 14].

**Figure 15:**
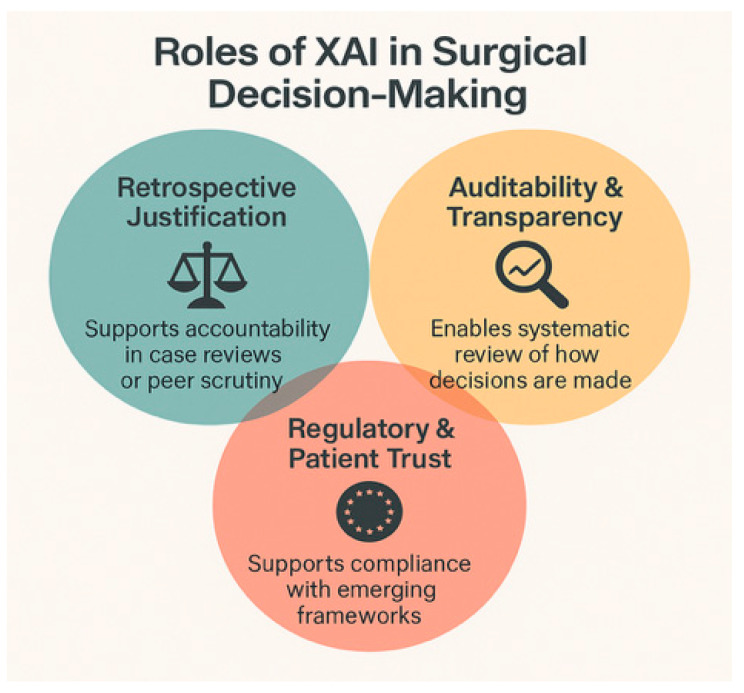
Roles of XAI in surgical decision-making. Retrospective justification—this is what supports accountability in case reviews, litigation, or peer scrutiny. Auditability and Transparency—this audit is crucial for both internal quality assurance and external regulation. Regulatory and Patient Trust—regulatory and patient trust alignment.

XAI plays a pivotal role in ethical justification and legal defensibility (auditability) of algorithmic decisions. SHAP and Grad-CAM are post hoc explanation methods that, as mentioned before, allow clinicians to trace which variables or image regions led to a specific decision: retrospective justification [44,45]. XAI is also a mechanism for auditability and transparency, enabling systematic review of how decisions are made, revealing sources of bias, error, or drift [28,36]. XAI supports compliance with emerging frameworks such as the European Regulation on Artificial Intelligence (EU AI Act) and the General Data Protection Regulation (GDPR), reinforcing the ethical imperative for transparency in high-risk medical decisions [46] [Figure 15].

In summary, surgical AI introduces complex liability questions involving surgeons, developers, and institutions. XAI strengthens ethical and legal safety nets by enabling clinicians to understand, justify, and document algorithmic decisions. Future governance will likely require shared accountability, built around transparent systems and clear clinician oversight.

### 4.2. Surgical Autonomy and Clinical Judgment

As AI becomes increasingly embedded in surgical environments, a fundamental ethical and clinical principle must remain central: AI must be considered as a support augmentative tool, operating within a framework that preserves clinical judgment, experience, and contextual reasoning; it must not be seen as a substitute for surgical expertise, nor an authority.

Surgeons possess a nuanced understanding of anatomy, physiology, intraoperative context, and patient-specific factors that no algorithm can fully replicate. Despite AI’s ability to generate predictions, it lacks true causal understanding, moral responsibility, and embodied experience [28,47]. Clinicians must interpret and challenge results within the clinical framework. Studies reinforce the importance of XAI, showing that when AI outputs are presented without context or explanation, surgeons may distrust the system or, conversely, defer its recommendations, risking automation bias [21,48].

One of the key contributions of XAI in surgery is enriching risk stratification tools and clinical decision trees. SHAP or LIME techniques are able to identify which patient-specific variables most influenced a model’s prediction, offering granular insights that can be integrated into evidence-based protocols [44,45]. For example, an explainable model predicting postoperative pneumonia might highlight smoking history, spirometry results, and operative time as key contributors, which enables the surgical team to tailor perioperative care and engage in shared decision-making with the patient, based on both evidence and transparency [49]. Furthermore, XAI facilitates selective trust, allowing surgeons to distinguish between high-confidence and low-confidence predictions. This is particularly valuable in complex or borderline cases, where surgical judgment must weigh algorithmic input against the unique clinical scenario [50].

As AI systems grow more sophisticated, alignment with clinician intent is becoming critical, and it is crucial to preserve the human element in surgical innovation: AI must not diminish human dimension of surgical care (e.g., empathy, ethical discernment, adaptability), and must serve as a cognitive interface that allows to understand and appropriately calibrate trust in AI suggestions [43,51].

### 4.3. Informed Consent and Shared Decision-Making

From preoperative risk calculators to RAS, AI is now embedded in multiple layers of surgical care: these tools inform and guide clinical choices, challenging traditional models of informed consent, and raising ethical concerns about autonomy and transparency [43]. Indeed, patients deserve, and have an ethical and legal right, to understand AI contributions to clinical decisions [37,38].

XAI pretends to facilitate patient–clinician dialogue. As mentioned before, GDPR mandates a ‘right explanation’ when decisions are made using automated processing, a principle that is increasingly relevant in high-stakes, data-driven surgical environments [46]. Beyond legal obligations, ethical standards require that patients be fully informed of any non-human elements influencing surgical decisions, particularly when those elements carry inherent uncertainty [49]. Evidence suggests that disclosure of AI involvement does not necessarily erode trust. Patients are more likely to reject care if they later discover AI involvement without their knowledge, especially when explanations are absent or opaque [52].

XAI will close the interpretability gap between complex models and human understanding. Clinicians are able to translate algorithmic decisions into clinically relevant explanations that patients can grasp, through techniques such as SHAP values, Grad-CAM visualizations, or prototype-based reasoning [29,53]. For instance, if a risk model predicts a 30-day postoperative mortality risk of 18%, SHAP-based explanations may show that age, lung function, surgical complexity, and nutritional status are the top contributing factors. This enables the surgeon to present these inputs in plain language, promoting shared decision-making based on both statistical insight and personalized care [54]. Instead of opaque recommendations, XAI empowers clinicians to narrate the logic behind AI outputs, making patients feel heard, respected, and involved [39]. In this way, XAI helps manage expectations and uncertainties, fostering trust, not only by making AI explainable to professionals, but also by making it communicable to laypersons (especially critical in surgical settings where patients must consent under stress or with limited time) [54].

Efforts to ensure consent is truly informed, contextualized, and patient-centered must consider not just what information is shared, but how it is delivered: XAI is crucial to enable this transformation [55]. XAI helps mitigate the gap in patients from disadvantaged backgrounds (language, literacy, age, education), who face greater difficulty understanding AI-based decisions, by offering visual and interactive explanations that enhance comprehension, reduce fear, and ensure inclusivity in the consent process [56].

### 4.4. Equity and Bias Detection

Surgical outcomes often reflect disparities across race, gender, socioeconomic status, geography, and disability [57]. Disparities stem from structural inequities in healthcare access, referral patterns, preoperative optimization, and intraoperative decision-making. Unfortunately, AI systems trained on historical data risk perpetuating or even amplifying these inequities, particularly when the datasets themselves are unbalanced or reflect biased clinical behavior [58]. Models trained primarily on data from high-resource hospitals may underperform when applied to underserved populations. Algorithms predicting surgical mortality or complications may reflect systemic under-documentation of symptoms or comorbidities in minority patients, leading to under-triage or misclassification [59].

XAI offers a promising avenue for the identification of biased patterns in model behavior. By attributing outcomes to specific input features (e.g., SHAP, LIME), clinicians and developers can identify when non-clinical factors such as insurance status, ZIP code, or race, disproportionately influence predictions [55]. SHAP analysis of a surgical risk model revealed that black patients were being systematically assigned lower priority for elective procedures: not due to physiological differences, but because of confounding variables correlated with race (e.g., incomplete lab work, delayed referral) [60]. Without explainability, such patterns might remain hidden inside black-box models. Algorithmic fairness audits are an emerging standard in clinical AI governance, which are supported by the use of XAI to audit performance across demographic subgroups, comparing how risk scores are distributed and interpreted for different populations [61].

Bias detection is only the first step toward fairer surgical algorithms. When unfair patterns are identified, developers and clinical leaders must act, either by rebalancing training data, removing confounding features, or adjusting thresholds for underrepresented groups [62,63]. Hospital and surgical departments must create feedback loops to ensure explainability leads to change, not just awareness.

Beyond bias detection, the integrity of clinical AI also depends on data provenance, reproducibility, and transparency throughout the AI pipeline [64]. Biased predictions frequently arise not only from imbalanced datasets but also from poorly documented data origins, opaque pre-processing decisions, and non-reproducible feature engineering pipelines. When the lineage of the training data (how, where, and by whom it was generated) is not explicitly tracked, it becomes difficult to contextualize model behavior, assess generalizability, or identify sources of structural bias. Transparent documentation of dataset composition, inclusion/exclusion criteria, missing-data handling, and temporal drift is essential for auditing both fairness and validity.

In this context, reproducible and open data collection practices play a critical role in building trustworthy surgical AI systems. Goulas et al. demonstrate how structured, automated data acquisition (e.g., reproducible web-scraping workflows) can enhance transparency, standardization, and replicability in medical research [64]. Their approach highlights that data pipelines can be systematically documented, version-controlled, and shared, enabling independent verification of datasets and reducing hidden sources of bias introduced during data collection. Although their application focuses on estimating international research collaboration, the underlying principles (explicit code availability, automated data extraction, timestamped data provenance, and transparent transformation steps) translate directly to clinical AI. Incorporating similar open-science practices into surgical AI development would allow institutions to more easily validate the integrity of input data, reproduce model-building steps, and detect where biases are introduced across the pipeline.

Ultimately, mitigating bias requires not only identifying inequitable model behavior through XAI but also ensuring that the entire data pipeline, from acquisition to pre-processing to model training, is transparent, reproducible, and open to audit [64]. Embedding reproducibility and data provenance standards into AI development strengthens fairness interventions, enhances interpretability, and supports trustworthy deployment across diverse surgical populations.

### 4.5. Trust and Team Dynamics

**Figure 16:**
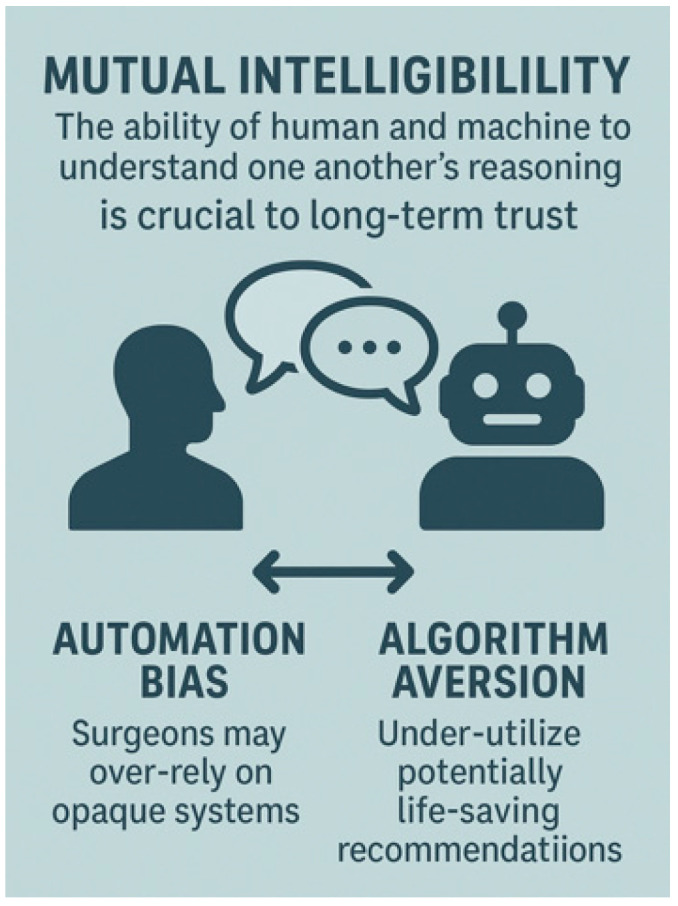
Mutual intelligibility in long-term trust. In high-stakes surgical contexts, lack of trust equates to non-use or misuse; surgeons need to see how the model arrived at a given decision (feature attribution, case comparisons, visual heatmaps).

Surgeons are trained to make rapid, high-consequence decisions based on a combination of evidence, experience, and intraoperative nuance. For surgeons to adopt AI tools, they must understand and be able to scrutinize the outputs: successful integration of AI in surgery depends not only on technical accuracy, but also on the development of trust, both individual and collective, among surgical team members [51,53,65] [Figure 16]. XAI helps establish cognitive congruence between algorithmic suggestions and surgical reasoning: if a decision support system recommends altering a resection plane based on real-time imaging data, a Grad-CAM visualization, or a case-based reasoning interface allows the surgeon to evaluate the logic behind the alert and judge its clinical relevance [29].

Trust in surgery is not limited to the primary operator. There is a distribution across a multidisciplinary team, which includes anesthesiologists, residents, nurses, perfusionists, and robotic support staff. AI systems are able to give intraoperative alerts (e.g., deviations in tissue perfusion, proximity to critical structures, or risk of surgical site infection). However, the entire team must understand what the system is flagging and why [66]. Each team member can contextualize the AI’s output relative to their role. XAI tools in RAS can help both surgeons and bedside assistants align their decisions in real time (e.g., XAI tools that show visual overlays of predicted anatomical boundaries) [34]. Furthermore, transparent AI supports smoother communication during high-pressure scenarios. Teams are more likely to heed AI-generated alerts or suggestions when those alerts are interpretable, consistent, and justifiable, rather than arbitrary outputs from a system perceived as obscure or unreliable [39].

Building institutional trust and training in AI fosters not only confidence in the tools but also a collective culture of learning and adaptation [67]. Regular postoperative debriefs involving AI recommendations, explained by XAI, allow surgical teams to evaluate accuracy, recalibrate expectations, and refine workflows [67]. XAI can be especially useful in teaching hospitals, for training surgical residents: it makes implicit decision-making processes explicit (e.g., comparing real-time AI alerts with attending surgeons’ decisions, and then reviewing SHAP or Grad-CAM explanations, can serve as valuable learning tools) [68].

## 5. Clinical Applications of Explainable AI in Surgery

### 5.1. Preoperative Phase-Risk Prediction

**Figure 17:**
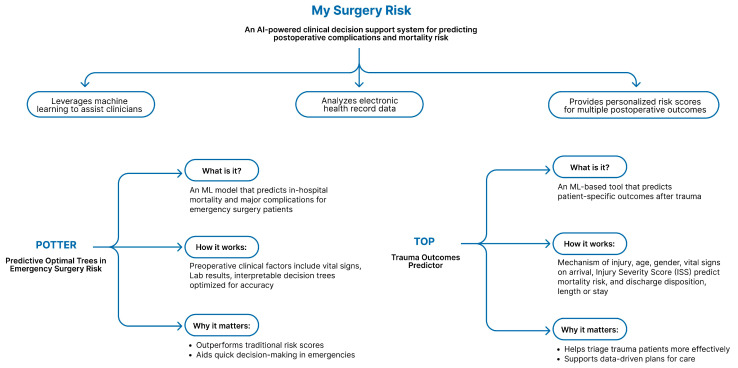
MySurgeryRisk is an AI-powered clinical decision support system developed by researchers at the University of Florida that leverages ML to assist clinicians in making more accurate, data-driven decisions before surgery; it predicts postoperative complications and mortality risk in surgical patients. POTTER is an ML model designed to predict in-hospital mortality and major complications for patients undergoing emergency surgery, using optimal decision trees that are easy to interpret yet highly accurate. TOP is an ML–based tool developed to predict patient-specific outcomes after trauma, such as motor vehicle crashes or falls. It is used to support trauma care and triage decisions.

The preoperative phase is critical for assessing surgical risk and optimizing patient outcomes. Large volumes of patient data (e.g., demographics, comorbidities, imaging, laboratory values, unstructured clinical notes) can be analyzed and synthesized by AI into individualized risk profiles (AI-powered risk calculators), surpassing the major challenges of traditional tools (e.g., manual data entry, and rigid structures), like American Society of Anesthesiologists (ASA) and the American College Society (ACS) National Surgical Quality Improvement Program (NSQIP) surgical risk calculators. This is making it possible to achieve high yield in the detection of high-risk patients [10,11,12,69]. A hospital-specific ML app applied in a real-world hip surgery study predicted adverse outcomes with an area under the receiver operating characteristic curve (AUROC) of 0.81, outperforming ASA-PS (0.63) [12]. University of Pittsburgh Medical Center (UPMC) gradient-boosted model, trained on 1.5 million patients, accurately predicted 30-day mortality and major cardiovascular events, deploying weekly evaluations directly within EHR workflows [69]. MySurgeryRisk, Predictive Optimal Trees in Emergency Surgery Risk (POTTER), and Trauma Outcomes Predictor (TOP) are examples of ML-based calculators, which outperform clinicians in assessing mortality and complications. These hospital-integrated web tools and XAI frameworks (e.g., MySurgeryRisk’s co-designed) are leading AI tools and systems [11,14,70] [Figure 17].

XAI-enhanced models have been increasingly employed to predict key outcomes (e.g., mortality, postoperative complications, need for ICU admission), being able to provide transparent explanations that reveal which patient-specific factors most influence risk predictions (e.g., age, lab values, comorbidities, physiological parameters) [67,71]. SHAP and LIME enable clinicians to dissect model outputs and understand the contributions of any risk variables [45,72]. For example, pneumonia is one of the most frequent respiratory postoperative complications. Risk factors, such as diminished lung function (e.g., low FEV1), smoking history, and pre-existing cardiac diseases, are considered high-predicted risk factors of postoperative pneumonia. In this way, surgeons will be able to personalize surgical planning by highlighting modifiable risk factors and anticipating complications. Choices about surgical approach, anesthesia, and perioperative optimization would also be better guided [44,53]. XAI will enhance surgical planning, shared decision-making, clinical confidence, and patient–clinician communication: surgeons will transparently explain to patients the rationale behind proposed management plans [54,73].

### 5.2. Intraoperative Phase

**Figure 18:**
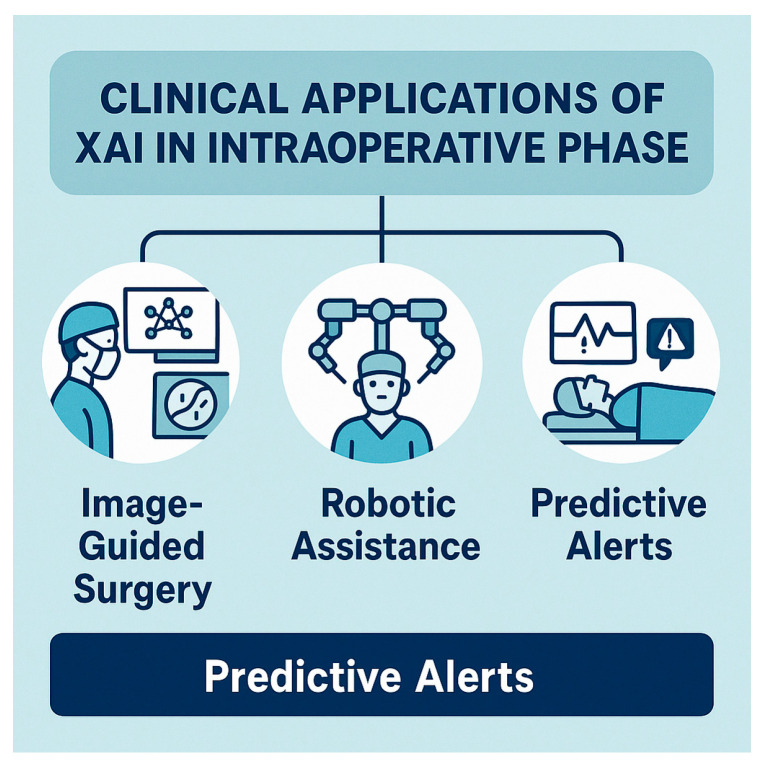
Clinical applications of XAI in the intraoperative phase: image-guided surgery, robotic assistance, and predictive alerts.

Clinical applications of XAI in the intraoperative phase are highly valuable [Figure 18]. For a surgery to be successful, accurate identification of critical anatomical structures and tumor margins is essential during the procedure. AI-based image segmentation tools applied to computed tomography (CT) and magnetic resonance imaging (MRI) can help identify tumor margins and vascular structures. XAI techniques will enhance real-time image-guided surgery by using computer vision techniques in order to assist surgeons in delineating tumor boundaries or vital tissues with greater precision [34]. Integration of explainability tools (Grad-CAM—Gradient-weighted Class Activation Mapping) in DL models, for instance, provides visual heat maps that highlight regions of interest on intraoperative imaging: this clarifies the model’s focus areas and enables surgeons to verify AI-driven guidance [44,74]. A recent scoping review of intraoperative video analysis in RAS esophagectomy (RAMIE) reported that AI models achieved ~84% accuracy in surgical phase recognition and anatomical landmark detection, underscoring the potential for real-time intraoperative decision support [75].

RAS with XAI-enhanced visual overlays increasingly leverages AI to improve dexterity, efficiency, and precision. RAS is enhanced by XAI through the generation of visual overlays that explain the rationale behind robotic movements or suggested instrument trajectories. Surgeons can easily understand why the robot proposes a specific action through such explanations: this allows them to maintain clinical control and confidence (e.g., when a robotic system adjusts dissection planes based on anatomical recognition, XAI methods can highlight the underlying imaging features or sensor inputs driving that decision, which facilitates real-time surgeon–robot collaboration) [21,28,76]. In the oncologic robotic surgery domain, a recent review described intraoperative AI support for tumor and vascular structure localization, margin assessment, and navigation, demonstrating how explainable models can help preserve healthy tissue and reduce warm-ischemia time [77].

With explainable indicators, intraoperative AI systems can provide predictive alerts to warn of impeding complications (e.g., hemorrhage, organ injury, procedural errors). By integrating explainability techniques, these alerts are accompanied by transparent, actionable indicators that specify why the alert was triggered (e.g., sudden changes in tissue perfusion metrics, instrument pressure, or unexpected anatomical displacement): this transparency enhances trust and allows a prompt and appropriate response by the surgical team, improving patient safety [34,67]. For example, in a general surgery review, AI was used for intraoperative task recognition and next-step prediction in robotic partial nephrectomy, highlighting the feasibility of anticipatory AI in the OR [78]. Alert fatigue and skepticism are real challenges that can limit their effectiveness.

### 5.3. Postoperative Monitoring and Outcomes

The postoperative phase is a vulnerable period during which early detection of complications (e.g., infections, anastomotic leaks, unexpected readmissions) is essential. AI-driven predictive models have demonstrated promise in forecasting these adverse events through analyzing complex patterns in EHR, lab values, vital signs, and other multimodal data [67,71]. Through algorithmic alerts, XAI helps clinicians validate risk factors, influence alerts, foster trust, and enable timely interventions. XAI will attribute predictive outputs to specific clinical features (e.g., elevated inflammatory markers, hemodynamic instability, comorbidities), and ultimately support more nuanced clinical judgment, helping avoid overreliance on black-box predictions. Ultimately, this indeed facilitates clinical validation [44,45].

Anastomotic leak is a devastating complication with high morbidity and mortality in postoperative patients, mainly in gastrointestinal surgery. Some ML models have been developed in order to predict leak risk, with explainable outputs for early detection, based on perioperative variables, intraoperative findings, and early postoperative data [59]. With XAI techniques, it will be possible to provide a transparent rationale for predicted risk, identifying key contributor factors (e.g., intraoperative blood loss, delayed return of bowel function, rising inflammatory markers), and enabling reduction in adverse outcomes [53].

Table 8 and Table 9 summarize the most relevant studies of XAI in surgery, and Table 10 summarizes the landscape of XAI in surgery. In summary, integration of explainability into postoperative AI monitoring systems allows surgical teams to confidently interpret alerts, personalize patient management, and optimize recovery pathways [54].

### 5.4. Training and Simulation

Training competent surgeons requires rigorous, objective, and structured assessment of technical and cognitive performance. AI can be applied in surgical skill assessment: traditional evaluation methods (e.g., global rating scales, subjective feedback) lack specificity and suffer from inter-rater variability.

AI-based assessment systems have emerged as powerful tools for automated skill evaluation in simulation and live environments. These systems are trained on annotated surgical video, kinematic data, and instrument tracking [88,89]. Explainable scoring systems for residents take these AI-based tools a step further by making the assessment criteria transparent. XAI-enhanced systems can highlight specific actions, instrument motions, or decision points that contributed to a low or high rating, enabling residents and educators to pinpoint areas for improvement: this will stop evaluation by assigning opaque performance scores [29].

Another particularity is the feedback mechanism using annotated video or instrument motion metrics. An AI system may detect poor bimanual dexterity or excessive motion economy during laparoscopic suturing. With XAI techniques (e.g., saliency maps, temporal attention models, or SHAP values), the system can visually or textually explain why a task was poorly scored (e.g., excessive wrist rotation, inconsistent needle angle) [90,91].

Modern surgical simulators and training platforms now incorporate real-time and post hoc feedback. XAI-enhanced feedback tools allow residents to see which specific segments of a procedure were flagged, the reasoning behind the flag, and how their performance compares to expert benchmarks [92]. This level of personalized and explainable feedback enhances the learning curve, encouraging deliberate practice and supporting competency-based education. Moreover, XAI systems can be valuable tools for faculty development and calibration, ensuring that teaching surgeons can also interpret and align their assessments with objective data [93]. By providing transparent, interpretable metrics, XAI supports fairness, motivates learners, and builds trust in AI-assisted evaluation: all key factors in surgical education [94].

## 6. Technical Considerations and Limitations

### 6.1. The Accuracy–Explainability Trade-Off

The integration of AI into surgical workflows introduces a crucial technical dilemma: how to balance model performance with interpretability. The accuracy–explainability trade-off is a particularly critical tension in high-stakes domains, like surgery: clinical decisions must be both data-driven and defensible.

AI models are divided into simpler models and black-box models [Figure 6]. Simpler models (e.g., logistic regression, decision trees, rule-based classifiers) allow clinicians to trace how inputs influence outputs. Their predictive performance falls short when dealing with complex, high-dimensional data such as imaging, real-time sensor streams, or multimodal EHR [95]. Differently, in surgery, high-performing black-box models (e.g., DNN, ensemble methods, gradient-boosted machines) might accurately predict postoperative complications or identify subtle imaging features, yet they offer little insight into how the prediction was made, limiting their clinical trustworthiness and adoption [72].

It is imperative to balance predictive power with clarity, which is not a binary trade-off. XAI is a growing field that aims to bridge this gap by applying post hoc interpretability techniques to high-performing models. SHAP, LIME, and Grad-CAM are XAI tools that allow surgeons to inspect specific predictions and assess whether the model’s reasoning aligns with established clinical knowledge [44,45]. Still, these explanations are not without limitations. Post hoc explainability cannot faithfully reflect the model’s internal logic, leading to a ‘plausible but incorrect’ narrative that can mislead users [96]. In surgery, false trust in explanations can be as dangerous as blind faith in predictions. Thus, XAI’s interpretability should be seen as a supplement to, and not a substitute for, critical human judgment [49].

XAI in surgery must perform in a context-dependent way, toward context-dependent solutions. For screening tools or patient-facing applications, higher explainability may be prioritized. For intraoperative systems where milliseconds matter, raw performance may take precedence, providing fail-safes and human override mechanisms in place [97]. Ultimately, the key is calibrated trust; it is crucial to ensure clinicians understand what AI does well, where it struggles, and how its predictions can be meaningfully integrated into clinical workflows [54].

### 6.2. Workflow Integration

**Figure 19:**
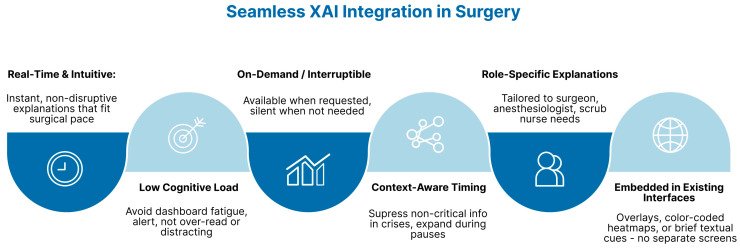
Design Principles for Intraoperative XAI Integration. One of the most significant barriers to adopting XAI in surgery (a high-stake environment) lies not in the algorithm itself, but in the challenge of seamlessly integrating its outputs into the real-time, high-pressure surgical workflow.

Surgical teams operate in time-sensitive, cognitively demanding settings, where any tool that introduces friction, distraction, or delay may be abandoned regardless of its analytical accuracy [98]. In this way, AI models must provide real-time, intuitive explanations, without compromising the user’s focus, timing, or situational awareness. Interfaces must be designed to support decision-making with minimal cognitive load, avoiding ‘dashboard fatigue’, or alert overload, which are known to hinder performance and reduce trust in clinical technologies [99] [Figure 19].

To be actionable intraoperatively, XAI’s inputs must be delivered via intuitive, real-time interfaces (e.g., overlays on endoscopic images, color-coded heatmaps, brief textual summaries). The aim is not to require the team to consult a secondary interface that may disrupt the procedure or increase mental workload; rather, it is to embed explainability into existing visual or auditory streams [67]. Complex graphs, or dense numerical tables, must be avoided [100]. In laparoscopic procedures, examples include boundary highlighting and predictive trajectory arcs embedded in the surgeon’s console or a heads-up display [101].

Explanations should be tailored to the surgical role: this is another often-overlooked consideration. ‘One-size-fits-all’ explanations do not apply to the numerous clinical roles and are counterproductive (e.g., a surgeon may require information on anatomical localization; an anesthesiologist might prioritize predictive alerts regarding hemodynamic instability; a scrub nurse may benefit from procedural step prediction for workflow anticipation) [89]. XAI outputs should be role-specific and tailored: each team member must be able to access the level and type of explanation supporting their function, without being overwhelmed by irrelevant data [53]. Modular interfaces or voice-assist prompts can deliver targeted XAI feedback based on a team member’s identity, task, or time point in the operation [54].

Context-aware AI systems recognize the phase of surgery and adapt the timing and modality of their explanations accordingly. These systems may offer an ideal compromise: they can suppress non-critical information during a crisis moment, or deliver full interpretability once a pause in the procedure allows for reflection [21]. Moreover, explanations should be available on demand when desired, but not continuously intrusive. This is called interruptible explanations. In order to preserve the usability and acceptability of AI tools in the operating room, these two principles are imperative.

### 6.3. Misleading Explanations

**Figure 20:**
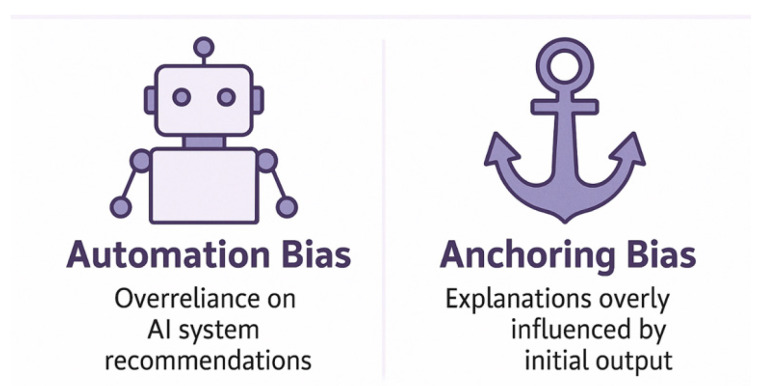
Automation Bias: when clinicians rely too heavily on AI, despite contradictory clinical evidence. When the explanation skews a team’s focus toward irrelevant features, while missing more important, but unmodeled factors, it is called anchoring bias.

It is important to note that many clinicians are not trained in ML theory and may not recognize that an ‘explanation’ is merely a proxy approximation of internal model behavior, and not a definitive clinical rationale [54]. Despite the principle of XAI being to make ML outputs more transparent, not all explanations are accurate, helpful, or safe. A growing body of research, mainly in the surgical sphere, is alerting to ‘Explanation fallacy’, defined as the idea that users may feel reassured by an explanation that appears plausible, especially when presented with visual or linguistic authority, giving a false sense of confidence, even if it does not accurately reflect the true behavior of the model. In the operating room, clinical decisions must be based on accurate, validated insights: this cognitive mismatch/misunderstanding can be dangerous, degrading decision quality. Superficially interpretable, but fundamentally misleading explanations must be avoided in surgery: this is a major ethical and technical concern, which can lead to overconfidence, automation, and anchoring bias, and inappropriate clinical actions [53,65]. Figure 20 explains the difference between automation and anchoring bias in XAI [53,65].

**Figure 21:**
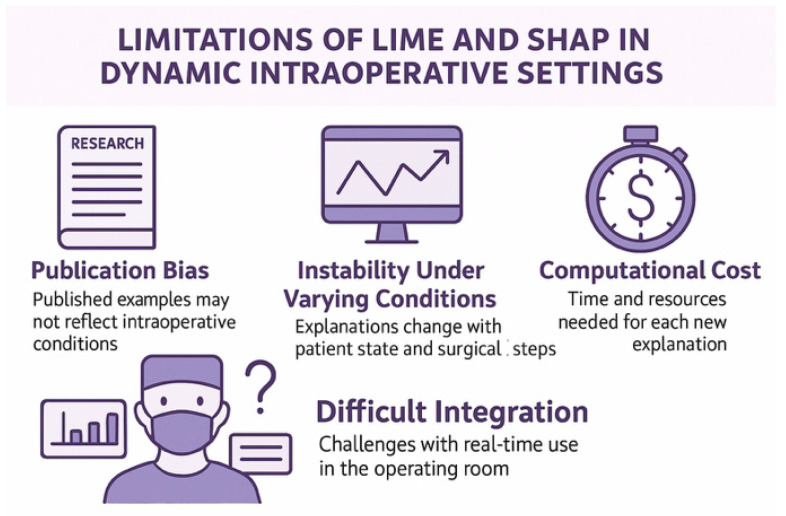
Limitations of LIME and SHAP in dynamic intraoperative settings.

When applied to the non-linear, time-sensitive, and multimodal data of surgical contexts, LIME and SHAP present challenges, despite offering localized, human-readable interpretations of individual predictions [54,96]. Critical moments in surgery, like dissection near vital structures or decisions to escalate to ICU postop, pose significant challenges to the use of LIME or SHAP-based explanations [Figure 21; Table 11] [54,96]. In order to mitigate these risks, some scholars advocate for robust and verifiable explanations: model-intrinsic interpretability, and rigorous validation of explanations themselves, not just the underlying model [95]. Others suggest human-in-the-loop systems, where expert oversight continuously tests the alignment between AI outputs, explanations, and surgical realities [97].

### 6.4. Data Heterogeneity and Generalizability

The high heterogeneity of surgical data (a broad range of procedures, techniques, devices, and patient populations) that varies not only across institutions but also across specialties, countries, and even individual surgeons is one of the most significant obstacles to deploying a reliable XAI in surgery [76]. Data sources in surgery include operative videos, instrument telemetry, EHRs, imaging, and anesthesia logs. These data are frequently stored in non-standardized formats, with variable quality and granularity, which complicates the training of robust AI models and the interpretation of their outputs via XAI frameworks [89]. An AI model trained to detect complications in colorectal surgery, using video from a high-resolution laparoscopic platform, may underperform when applied to general surgery, using a different imaging system, or in a center with different clinical protocols. In this way, explanations generated by XAI tools may then become misleading or invalid, as they reflect context-specific patterns and are not generalizable across sites. An explanation derived from a model trained in one institution’s dataset may highlight features (e.g., surgical timing, blood pressure thresholds) that do not apply elsewhere, due to differing protocols, instrumentation, or patient demographics: the generalizability problem [49].

**Figure 22:**
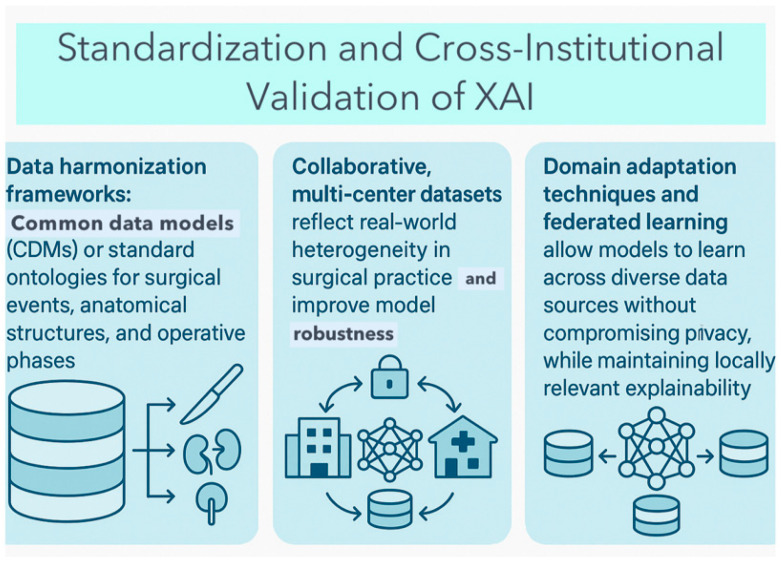
Standardization and Cross-institutional validation of XAI tools.

Thus, XAI tools must undergo rigorous external validation across diverse surgical settings to ensure their interpretability remains clinically meaningful, and not merely technically consistent [54]. A model that can explain its predictions well in one hospital may produce plausible, but incorrect explanations in another, if trained on biased or narrowly representative data [59]. To address this challenge, there is growing advocacy for standardization and cross-institutional validation [Figure 22]. Explainability methods themselves should also be subject to validation metrics across environments (fidelity, stability, consistency), not just the models they accompany.

## 7. Regulatory and Institutional Context

### 7.1. Global Regulatory Landscape

**Figure 23:**
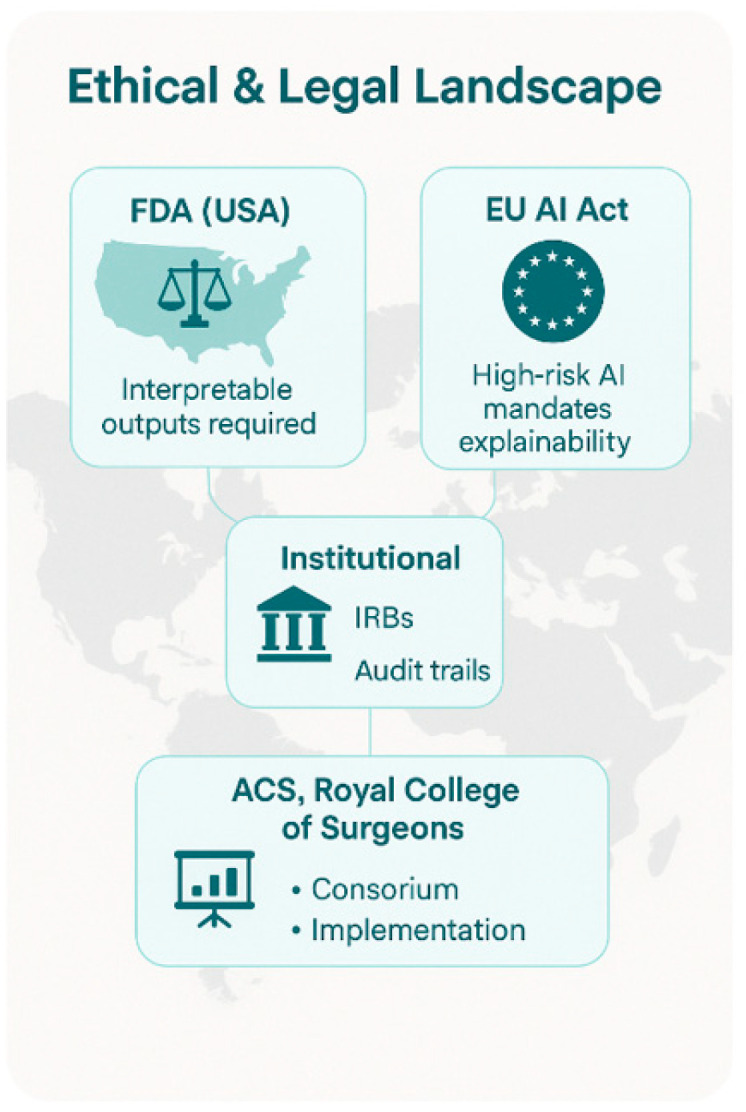
Evolving global regulatory, ethical, and legal landscape, and emerging institutional governance mechanisms. ACS—American College of Surgeons; EU AI Act—European Union Artificial Intelligence Act; IRBs—Institutional Review Boards; FDA—Food and Drug Administration.

Because AI and XAI continue to permeate surgical environments, the need for coherent regulatory, ethical, and institutional oversight frameworks is becoming increasingly urgent. High-stakes clinical contexts—such as robotic surgery, risk prediction, and intraoperative decision support—demand both performance and accountability. This section outlines the evolving global regulatory landscape, emerging institutional governance mechanisms, and professional guidelines shaping the integration of XAI into surgical practice [Figure 23].

AI-based devices must be interpretable for clinical use. In the United States, the FDA has begun to formalize expectations for transparency in AI-enabled medical devices. While early AI applications were approved under traditional device frameworks, newer “adaptive” or continuously learning algorithms have prompted the FDA to release proposed regulatory frameworks requiring these systems to be interpretable and clinically actionable for end users [102]. The FDA’s 2021 discussion paper, “Artificial Intelligence and Machine Learning Software as a Medical Device (SaMD)”, emphasizes the need for clear, context-specific information about how the algorithm works to support both informed consent and safe clinical use [103].

The European Union Artificial Intelligence Act (EU AI Act), currently moving through final legislative phases, categorizes AI systems used in healthcare, and particularly in surgery, as “high-risk” applications. This designation requires that such systems comply with robust transparency, human oversight, and accountability requirements [41]. For surgical AI tools, it is mandatory to provide explanations that are intelligible to non-technical users (clinicians and patients) [104]. Failure to provide adequate explainability or auditability could lead to restrictions or bans on deployment within EU member states.

Table 12 summarizes how different study designs in surgical AI research align with established evaluation frameworks and the regulatory expectations emerging from the EU AI Act, GDPR, and medical-device quality systems. Each study type, from early model development to post-market monitoring, maps to a specific set of methodological standards (e.g., TRIPOD-AI, DECIDE-AI, SPIRIT-AI, CONSORT-AI, IDEAL) that guide transparent reporting, risk mitigation, and reproducibility. For each stage, the table identifies XAI artifacts that should be archived to ensure traceability of model behavior, support auditability, and enable retrospective verification of clinical decision pathways. These artifacts include saliency maps, attribution vectors, model cards, explanation logs, and clinician–AI interaction metadata, depending on the phase of evaluation. This checklist also outlines how explanation assets intersect with legal and regulatory obligations. Under the EU AI Act, high-risk clinical AI systems must maintain robust documentation of model versions, input data provenance, interpretability methods, and human-oversight mechanisms. GDPR principles, mainly data minimization, purpose limitation, and pseudonymisation, shape what explanation outputs can be stored, and how they must be handled. Medical-device development frameworks (Table 13) require traceability of model design, change management, software lifecycle documentation, and usability evaluations, all of which are strengthened by systematic archival of XAI outputs. Together, these mappings provide a unified structure for researchers to design studies that are technically rigorous, ethically compliant, and aligned with regulatory expectations. They also offer a practical guide for documenting XAI pipelines in ways that facilitate clinical implementation, external inspection, and long-term system monitoring. Table 13 summarizes frameworks and regulatory references.

### 7.2. Institutional Ethics and Oversight

**Figure 24:**
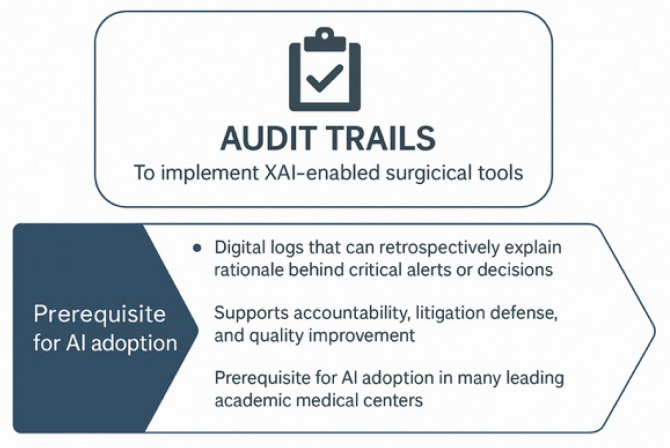
Audit trails support accountability, litigation defense, and quality improvement, and are becoming a prerequisite for AI adoption in many leading academic medical centers.

Institutional Review Boards (IRBs) and hospital AI oversight committees (local level) recognize that opacity in AI systems can obscure bias, mask errors, and erode trust, particularly when the outputs influence high-stakes surgical decisions. They require that clinical AI systems undergo evaluation not only for safety and efficacy, but also for explainability [Figure 23] [49]. To implement XAI-enabled surgical tools (e.g., predictive analytics platforms, robotic guidance systems), it is mandatory that these systems include audit trails [Figure 24] [21,67,103].

### 7.3. Guidelines from Surgical Societies

Professional societies have demonstrated an emerging interest in ethical and operational frameworks for integrating AI and XAI into surgical practice. For instance, the American College of Surgeons (ACS) has published discussion papers emphasizing the importance of transparency, human oversight, and patient-centered design in AI adoption [105]. The Royal College of Surgeons (RCS) has emphasized the risks of algorithmic harm if systems are poorly understood, noting the importance of multidisciplinary governance models and clinician involvement in AI validation and deployment. The Society of Thoracic Surgeons (STS) and the European Society of Thoracic Surgeons (ESTS) are beginning to integrate AI into quality registries and performance metrics, which may soon include requirements for explainability. Audit trails are mandatory [Figure 23 and Figure 24] [21,103].

It is of utmost importance to integrate XAI standards into credentialing frameworks, surgical training curricula, and quality improvement programs. In the future, demonstration of competency in surgical techniques and in the interpretation of AI-assisted decision systems may be required for board certification and hospital privileging [89].

### 7.4. AI Model Validation

Many AI algorithms in medicine are trained purely to optimize predictive accuracy (association), but then implicitly or explicitly imply intervention effects. Without Karamitro et al.’s concise and practical framework for assessing causality in medical AI research, summarized in Table 14, this can be misleading [106]. For clinical AI tools’ validation, it is insufficient to rely solely on statistical associations: for a system to support intervention decisions, one must evaluate whether the prediction reflects a causal mechanism. By embedding the six steps and decision-points into AI model development and evaluation, researchers can enhance the trustworthiness of deployment claims and reduce the risk of inadvertent harm stemming from misinterpreted associations. In other words, evaluating whether an AI model’s outputs can be interpreted, or safely deployed, as causal claims rather than mere statistical associations could be performed.

In the context of an AI system, relying on high-accuracy predictions does not guarantee that changing the input, or applying the model, will have the intended effect in real-world care: observational data often mask reverse causality, confounding, or selection bias [106]. Incorporating causal inference methods into AI validation strengthens the system’s explainability and trustworthiness, helping ensure that, if the model is deployed, the underlying assumptions, data-generating mechanisms, and organizational context align with the causal claims [106]. An AI model might find that postoperative complication rates are higher in patients who had longer operative times. Without careful causal framing, it might be suggested “to reduce operative time, and complications will drop”, ignoring confounding variables (e.g., sicker patients tend to have longer times). Validation with causal thinking might instead reveal that time is a proxy variable, not an actionable cause.

## 8. Future Directions and Recommendations

### 8.1. Human-Centered XAI Design

**Figure 25:**
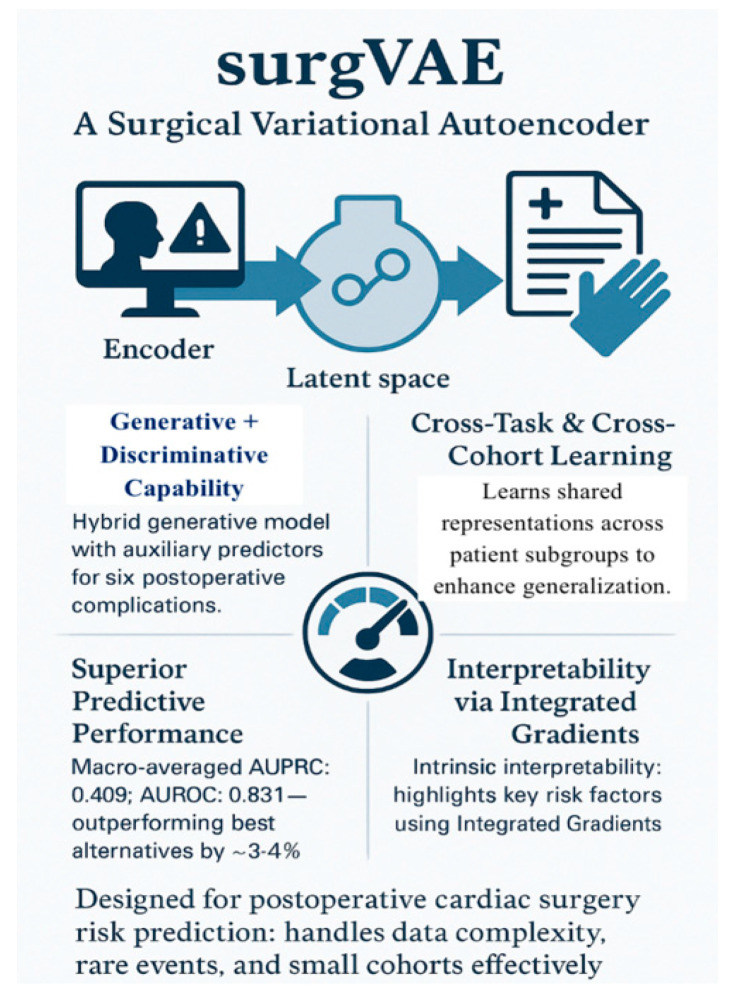
Key features of surgVAE. surgVAE integrates a variational autoencoder with auxiliary complication predictors to model six postoperative risks after cardiac surgery. Cross-task and cross-cohort learning improve generalization, achieving strong performance (AUPRC = 0.409; AUROC = 0.831). Integrated Gradients provide patient-level interpretability.

The future holds newer AI innovations: multi-task learning models in predicting postop complications for cardiac surgery (e.g., surgVAE) [Figure 25]; and human-centered co-design tools built with clinician input, and focused on usability, applicability, and trust calibration (e.g., MySurgeryRisk) [107,108].

As XAI matures within the sphere of surgical care, future progress will depend on human-centered design, rigorous validation, and cultural transformation in clinical education to ensure that interpretability supports decision-making. Human-centered design (HCD) principles recommend tailoring explanation formats by user role, cognitive load, and task specificity (20). Personalizing explanations by user role (surgeon, anesthesiologist, nurses, trainees, patients) will surpass static ‘one-size-fits-all’ outputs, and will allow explanations with varying depth, format, and timing [53]. Surgeons may need anatomically localized visual feedback, while patients require simplified language for informed consent, and residents benefit from pedagogical justifications.

Participatory co-design with end-users in the operating room is essential to ensure relevance and usability. Clinicians must be engaged in iterative prototyping, feedback cycles, and scenario testing. This strategy has been shown to improve the acceptance and real-world performance of AI tools [109]. These methods should become standard during the development of surgical XAI platforms.

### 8.2. Real-Time and Interactive XAI

Real-time AI outputs must integrate seamlessly into the operative workflow without increasing cognitive burden. Future systems will need to deliver ‘on-demand’ explainability, triggered by context or user query, rather than displaying continuous, intrusive outputs. Emerging strategies include attention-sensitive interfaces, minimalist overlays, and low-latency summary prompts embedded into robotic consoles or endoscopic displays [89].

Advanced interfaces (such as augmented reality, head-mounted displays) may present XAI explanations via spatial cues (e.g., color-coded tissue margins or dynamic trajectory indicators). It is expected that voice-assisted AI agents might deliver context-aware verbal explanations during procedures, without requiring visual attention shifts [92]. However, these modalities must undergo human factors testing to ensure they enhance, rather than distract from, clinical performance.

### 8.3. Validation, Benchmarking, and Reporting

**Figure 26:**
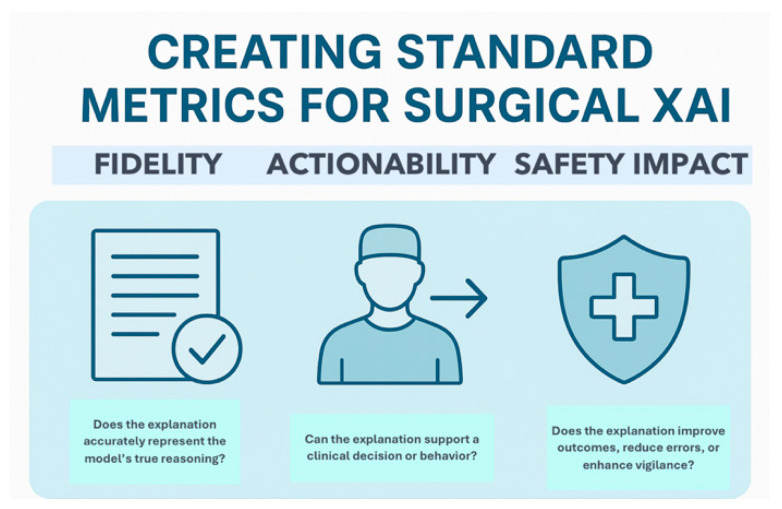
Pillars for creation of standard metrics in surgical XAI: Fidelity, actionability, and safety impact.

Transparent documentation of how explanations are generated is mandatory. AI urgently needs validated, domain-specific benchmarks to assess the quality and safety of XAI methods [44]. Fidelity, actionability, and safety impact are the proposed criteria, described in Figure 26. Frameworks like DECIDE-AI were initially proposed for early-phase AI evaluation in healthcare: they should be extended to include XAI-specific reporting items [109].

In order to improve trust, reproducibility, and regulatory compliance, especially in high-risk clinical decisions, future surgical AI systems should include machine-readable and clinician-friendly documentation describing how explanations are generated, their known limitations, and data dependencies [49].

### 8.4. Education and Skills Development

**Figure 27:**
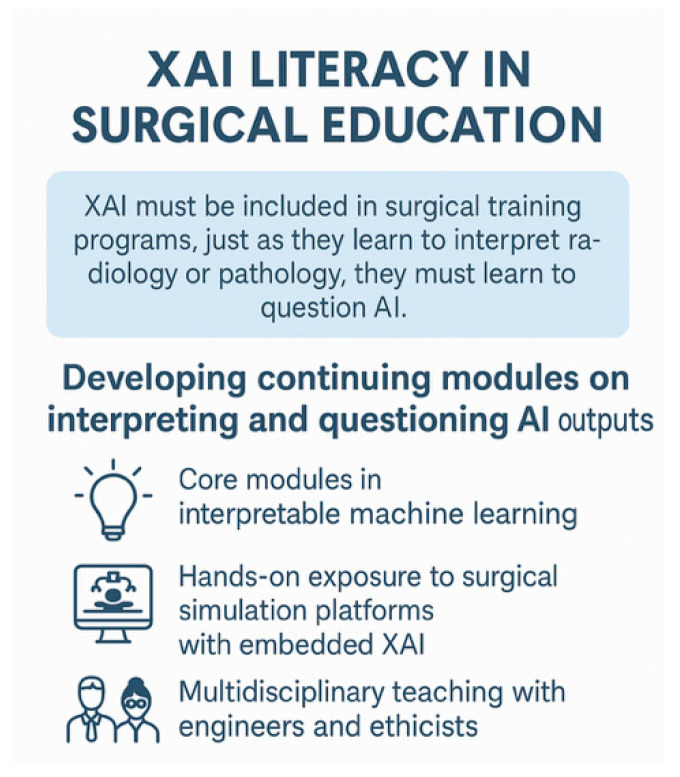
Education and skills development. Continuing medical education might include topics like interpreting risk stratification tools, recognizing misleading explanations, and interacting with intraoperative decision support systems.

XAI literacy must be included in surgical training programs; just as they learn to interpret radiology or pathology, they must learn to question AI. Surgical trainees must develop foundational literacy in AI and XAI, including an understanding of model biases, types of explanations, and how to critically appraise AI-generated recommendations [90] [Figure 27].

Developing continuing modules on interpreting and questioning AI outputs, programs should include: core modules in interpretable ML, hands-on exposure to surgical simulation platforms with embedded XAI, and multidisciplinary teaching with engineers and ethicists. For current practitioners, continuing medical education (CME) offerings should include short courses, webinars, and certifications on XAI in surgery [21]. Only through ongoing education can XAI be safely integrated into everyday clinical judgment.

## 9. Discussion

Surgery mostly relies on image analysis, and the role of Generative AI in diagnosis (especially in image interpretation) remains experimental, lagging behind specialized CNNs, which are more accurate in image analysis. Despite the recent multimodal capabilities of ChatGPT-4 and Generative AI, particularly LLMs, its current diagnostic performance and accuracy for image interpretation are suboptimal. It is mandatory to further refine and improve, with domain-specific tuning, before LLMs can be used as tools for image interpretation.

Integration of AI into surgical care offers transformative potential, but its success hinges on more than technical performance. XAI is essential for ensuring that these technologies are safe, fair, and aligned with the core values of clinical practice. By making algorithmic decisions transparent and interpretable, XAI fosters trust, enhances accountability and safety, and supports informed, patient-centered decision-making. However, explainability is a key challenge in Generative AI because of its ‘black-box’ nature: Generative AI in medicine will require robust XAI to ensure clinicians understand why an AI suggested or generated a particular interpretation, reducing risks of automation bias, and thus supporting accountability.

## 10. Conclusions

For AI to be truly effective in surgery, accuracy must be paired with explainability: a requirement that becomes even more critical in high-stakes, time-sensitive clinical environments. As surgical teams increasingly interact with complex AI systems, the ability to understand and question these outputs will define both clinical safety and ethical defensibility.

Crucially, the future of surgical AI will depend on interdisciplinary collaboration: bringing together surgeons, engineers, ethicists, and legal experts to design tools that empower, rather than replace, surgical expertise. Only through this shared effort will it be possible to build AI systems that enhance surgical performance, while preserving professional judgment, patient trust, and medical accountability.

## Figures and Tables

**Table 1:** Subtypes of Generative AI. Generative AI refers to a class of algorithms capable of producing new content (e.g., text, images, audio, or even synthetic patient data), based on patterns learned from large datasets. Unlike traditional discriminative models that classify or predict outcomes, generative models are designed to create novel outputs that mimic the underlying data distribution. GANs—Generative Adversarial Networks; LLMs—Large Language Models; VAEs—Variational Autoencoders.

Types of Generative AI		Applications in Medicine
LLMs	Built primarily on transformer architectures (e.g., GPT-4). Trained on massive corpora of text, capable of generating coherent text, answering questions, or analyzing multimodal data (text + images).	Used for drafting reports, summarizing guidelines, providing decision support, and increasingly for exploratory image interpretation
GANs	Consist of a generator and a discriminator in competition.	Widely used in medical imaging for data augmentation (e.g., creating synthetic radiographs or histology slides), improving resolution, and reducing noise.
VAEs	Encode input into a latent space and reconstruct outputs.	Useful in anomaly detection and simulation of patient-specific scenarios (e.g., disease progression models).
Diffusion Models (Stable Diffusion, DALL.E-like systems)	Gradually denoise random noise to generate high-quality images.	Potential in medical imaging for reconstructing or enhancing diagnostic images.

**Table 2:** Studies on ChatGPT in Clinical Diagnosis. AUROC—Area Under the Receiver Operating Characteristic Curve; CNN—Convolutional Neural Network; CT—Computed Tomography; GERD—Gastroesophageal Reflux Disease; GPT—Chat Generative Pre-Trained Transformer; NSCLC—Non-Small Cell Lung Cancer.

Study/Year	Domain	Design/Setting	Performance	Agreement/Additional Findings
Hirosawa et al. [3] Exploratory Internal Medicine Vignettes (2023)	Internal Medicine	Complex clinical vignettes; evaluated diagnostic accuracy	ChatGPT-4: 83% (10 differential diagnoses); 81% (5 differential diagnoses); 60% (final diagnosis)—comparable to internal medicine specialists	Comparable to human specialists in diagnostic reasoning
Sonoda et al. [4] Radiology Cases with ChatGPT-4	Radiology	Case-based differential diagnosis task	49.4% accuracy for the three-item differential diagnosis list	Suboptimal performance vs. expected radiology diagnostic standards
Henson et al. [5] GERD Recommendations (2023)	Gastroenterology (GERD)	Evaluation of ChatGPT recommendations for GERD management	90% of chatbot recommendations were appropriate	Demonstrated safety and reliability in >90% of cases
Gorelik et al. [6]—Pancreatic Cystic Lesions (2024)	Gastroenterology (Pancreatic Cysts)	Customized GPT tested on 60 clinical scenarios	87% adequate recommendations (52/60 scenarios); comparable to experts	High agreement with gastroenterologists
Dehdab et al. [8] Chest CT Interpretation (2024)	Diagnostic classification of chest CT scans	60 CT scans covering COVID-19, NSCLC, control cases	56.8% accuracy CNNs (literature: >80–90%)	Suboptimal performance; below clinically acceptable standards
Shifai et al. [9] Dermatoscopy (2024)	Dermatology	50 cases Differentiating melanoma from benign nevi	Poor discrimination: accuracy not clinically adequate CNNs (State-of-the-Art: AUROC > 0.90)	Chat-GPT failed to reach acceptable thresholds; CNNs outperform significantly

**Table 3:** LLMs versus CNNs—primary function, strengths, weaknesses, clinical adoption, and role in Generative AI. CNNs are able to analyze spatial data, such as images, because the layout of these neural networks resembles the animal visual cortex, by processing information across multiple layers—this allows for the extraction of numerous features.

Feature	Large Language Models (LLMs)	Convolutional Neural Networks (CNNs)
Primary Function	Text generation, reasoning, multimodal input handling (text + image)	Image classification, segmentation, and feature extraction
Strengths	Natural language explanations; integration of text and image; flexible reasoning	High diagnostic accuracy in image tasks; robust for radiology, pathology, dermatology
Weaknesses	Suboptimal diagnostic accuracy in image interpretation; hallucinations	Less interpretable; limited to the image domain; not designed for narrative reasoning
Clinical Adoption	Early exploratory phase; performance inconsistent	Already integrated into workflows (radiology triage, dermatology lesion detection, pathology slide analysis)
Role in Generative AI	Text-to-image analysis and multimodal reasoning	Core architecture for image analysis; benchmark for performance

**Table 4:** Key applications of Explainable Artificial Intelligence (XAI) in Surgery.

Key Applications of XAI in Surgery	
Preoperative planning	Explainable models pinpoint critical variables, driving risk assessments: ✓ Anatomical ✓ Laboratory
Robotic and image-guided surgery	XAI techniques allow surgeons to understand AI-driven anatomical segmentation or instrument planning: ✓ Visual overlays ✓ Interactive interfaces
Postoperative monitoring	Transparent alerts help care teams: ✓ Understand deterioration predictions ✓ Differentiate between false alarms vs. clinically meaningful signals

**Table 5:** Future directions of Explainable Artificial Intelligence (XAI) in Surgery.

Ongoing and Future Directions of XAI	
Adaptive and multimodal explanations	Tailored to clinicians’ specialties and contexts.
Federated Learning and Privacy-preserving XAI	Enabling trustworthy collaboration across institutions.
Human AI co-design	Bringing together surgeons, ethicists, developers, and patients. Ensure tools align with clinical needs and values.

**Table 6:** Examples of Explainable Artificial Intelligence Techniques, used in Surgery.

XAI Technique	Application in Surgical Context	Strengths and Limitations
SHAP	Preoperative risk models	Clear feature weights, but computationally heavy
LIME	Local explanations of model outputs	Simple instance-level reasoning, but unstable
Grad-CAM	Imaging tasks: segmentation, tissue localization	Intuitive visual maps; needs complementing methods
Saliency Maps	Fine-grained pixel-level highlighting	Too noisy alone Best when combined with CAM
Case-based reasoning	Prototype comparison in decision support	Clinically meaningful, but needs curated databases
Overlay XAI in robotics	Real-time anatomy annotation	Intuitive for surgeons, technical integration required

**Table 7:** Core Ethical dimensions of XAI in surgery.

Ethical Dimension	Explanation	Implications for Surgical Practice
Transparency	The degree to which an AI system’s decision-making processes can be understood by humans.	Surgeons must be able to understand how AI systems reach conclusions to ensure safe and reliable use.
Accountability	Clear identification of responsibility for AI-driven decisions and outcomes.	Ensures liability is traceable—critical in case of error or adverse events.
Fairness and Bias Mitigation	Ensuring AI does not perpetuate or amplify health disparities through biased data or models.	Promotes equity in surgical care across patient demographics and clinical contexts.
Autonomy and Informed Consent	Respecting patient autonomy by enabling clinicians to explain AI-supported decisions to patients for shared decision-making.	Enhances trust and ensures ethically valid consent processes.
Safety and Reliability	Guaranteeing that AI models perform consistently and robustly, especially in high-risk settings like surgery.	Reduces the risk of harm from erroneous or unstable AI recommendations.
Privacy and Data Protection	Ensuring patient data used in training and deploying AI models is handled ethically and complies with regulations (e.g., GDPR).	Maintains trust and adheres to legal standards in AI development and application.
Professional Integrity	AI should support—not replace—clinical expertise, preserving the surgeon’s professional judgment and decision-making autonomy.	Encourages human oversight and prevents overreliance on “black-box” tools. XAI is expected to be a technical solution, and at the same time a philosophical safeguard, thereby enhancing, not undermining, surgical autonomy.
Education and Literacy	Ethical deployment of AI requires clinicians to be adequately trained in understanding and interpreting AI tools.	Promotes safe integration and helps clinicians challenge AI recommendations when appropriate.

**Table 8:** Studies of explainable artificial intelligence in surgery.

Year	Journal	Study/First Author	Surgical Domain	Task	XAI Method(s)	N/Dataset	Study Type	Design (Retro/Prosp)	Centers (Single/Multi)	**External Validation**	**Prospective Data Collection**	**Human-Factors/Usability**
2025	npj Digital Medicine	Riva-Cambrin et al. [79]	General (robotic and endoscopic video)	Task and skill classification with transparent ‘liquid white box’	White-box design + interpretable features	Surgical video datasets	Methodology + validation	Method + retrospective	Multi-dataset (video)	No/Unclear	No/Unclear	No
2024	JCO Clinical Cancer	Hernandez et al. [80]	Oncologic surgery (inpatients with cancer)	Preoperative prediction of postoperative complications	SHAP-based explanations	Single health system inpatients with cancer	Model development and validation	Retrospective EHR	Single health system	No/Unclear	No/Unclear	No
2024	Surgical Endoscopy (open via PMC)	Lopez-Lopez et al. [81]	Hepatobiliary (laparoscopic liver resection, segments 7–8)	Predict surgical complexity, outcomes, and conversion to open	SHAP (global and local)	585 pts, 19 hospitals (international)	International multicenter study	Retrospective (international registry)	Multicenter (19 hospitals)	No/Unclear	No	No
2025	Journal of Thoracic Disease	Wang et al. [82]	Thoracic surgery	Predict postoperative pulmonary complications	Explainable ML (e.g., SHAP algorithm)	Retrospective cohort	Model development and validation	Retrospective cohort	Single-center (per article)	No/Unclear	No	No
2022	International Journal of Surgery	Fransvea et al. [83]	Emergency general surgery (elderly)	Predict 30-day postoperative mortality	Interpretable ML models (reporting feature effects)	FRAILESEL multicenter registry (Italy)	Prospective cohort secondary analysis	Retrospective	Multicenter (Italy)	No/Unclear	Yes (prospective registry)	No
2022	JAMA Network Open	Deng et al. [84]	Surgical oncology (cytoreductive surgery)	Predict major postoperative complications	SHAP (feature attribution, dependence plots)	Multicenter CRS cohort	Development and external validation	Retrospective development + external validation	Multicenter	Yes	No	No
2021	Scientific Reports	Zeng et al. [85]	Pediatric cardiac surgery	Predict postoperative complications using intraop BP + EHR	SHAP (global + patient-level)	1964 pts (single center, China)	Model development and internal validation	Development/benchmarking	Single-center	No/Unclear	No	No
2023	Sensors	Arabian et al. [86]	General laparoscopic (video)	Phase recognition with attention module (P-CSEM)	Attention maps/saliency for interpretability	Cholecystectomy datasets	Method + benchmarking	Development/benchmarking	Single-center/Unclear	No/Unclear	No	No
2022	Surgical Endoscopy (Springer)	Shinozuka et al. [87]	Hepatobiliary (lapcholecystectomy)	Surgical phase recognition from endoscopic video	Post hoc visualization (e.g., saliency/attention)	LC videos	Model development	Method (video)	Single-center/Unclear	No/Unclear	No	No

**Table 9:** Latency, Effect Size, Precision–Recall, Validation Type, Human Factors Assessment, and Intraoperative Relevance of the studies cited in Table 8.

Year	Journal	Study/First Author	Surgical Domain	Human Factors/Usability	Latency	Effect Size	Precision–Recall	**Validation Type**	**Human-Factors Assessment**	**Intraoperative Relevance**
2025	npj Digital Medicine	Riva-Cambrin et al. [79]	General (robotic and endoscopic video)	No	Not reported (video inference latency seldom reported)	Not reported	Not reported	Internal (multi-dataset)	No HFE evaluation	High (direct intraop video analysis)
2024	JCO Clinical Cancer	Hernandez et al. [80]	Oncologic surgery (inpatients with cancer)	No	Not reported	Not reported	May include class-specific precision/recall (not PR-AUC)	Internal only	Not evaluated	Low (preoperative only)
2024	Surgical Endoscopy (open via PMC)	Lopez-Lopez et al. [81]	Hepatobiliary (laparoscopic liver resection, segments 7–8)	No	Not reported	Some ORs for risk (depending on model output)	Precision sometimes reported; PR-AUC not typical	Internal multicenter	Not evaluated	Moderate (planning but not intraop)
2025	Journal of Thoracic Disease	Wang et al. [82]	Thoracic surgery	No	Not reported	Not reported	Not reported	Internal only	Not evaluated	Low
2022	International Journal of Surgery	Fransvea et al. [83]	Emergency general surgery (elderly)	No	Not reported	Not reported	Possibly precision/recall	Internal multicenter	Not evaluated	Low–Moderate (preop decision-making)
2022	JAMA Network Open	Deng et al. [84]	Surgical oncology (cytoreductive surgery)	No	Not reported	ORs are often reported in CRS literature	May include class precision/recall	Internal + External	Not evaluated	Low
2021	Scientific Reports	Zeng et al. [85]	Pediatric cardiac surgery	No	Not reported	Not reported	Not reported	Internal	Not evaluated	Moderate (intraop features used)
2023	Sensors	Arabian et al. [86]	General laparoscopic (video)	No	Not reported	Not reported	Typically report F1; PR-AUC rarely	Internal	Not evaluated	High
2022	Surgical Endoscopy (Springer)	Shinozuka et al. [87]	Hepatobiliary (lapcholecystectomy)	No	Not reported	Not reported	Not reported	Internal	Not evaluated	High

**Table 10:** Landscape of explainable AI in surgery. Phase—indicates the point along the surgical care continuum in which the AI system operates (preoperative tasks involve risk assessment and planning; intraoperative tasks involve real-time video or sensor interpretation; postoperative tasks focus on surveillance and outcome prediction; and cross-phase systems integrate data across multiple stages). Task Category—specifies the clinical or technical function being performed by the AI model, such as risk prediction, surgical phase recognition, workflow analysis, or complication forecasting. Model Types Used—lists the predominant machine learning or deep learning architectures employed for the task, including tabular ML algorithms (e.g., XGBoost), convolutional neural networks (CNNs), recurrent/temporal models, transformers, graph neural networks, or multimodal architectures. XAI Techniques—describes the interpretability approaches used to explain model behavior (including feature-attribution methods as SHAP, attention-based transparency, saliency/Grad-CAM visualization, rule-based surrogates, or concept-based explanations). Key Metrics Reported—summarizes the core performance metrics typically used in each phase and task; metrics include discrimination measures (e.g., AUROC, AUPRC), classification performance (accuracy, F1, mAP), calibration (Brier score), effect sizes (odds ratios, relative risks), and system-level metrics, such as latency or frame-level error, when reported.

Phase	Task Category	Model Types Used	XAI Techniques	Key Metrics Reported
Preoperative	Risk prediction, triage, mortality prediction, complication prediction, and surgical complexity scoring	Tabular ML (GBM, RF, XGBoost), Logistic regression, Transformer models for EHR	SHAP (global/local), feature importance, partial dependence, counterfactuals (rare)	AUROC, AUPRC (rare), calibration (Brier), OR/RR, effect size, sensitivity/specificity
Intraoperative	Phase recognition, workflow analysis, skill assessment, anomaly detection, video classification	CNNs, 3D-CNNs, LSTM/GRU, Vision Transformers, “liquid white box” architectures	Saliency maps, Grad-CAM, attention maps, embedded transparency, spatial–temporal attribution	Accuracy, F1, mAP, latency (rare), frame-level error, PR-AUC (rare)
Postoperative	Complication forecasting, length-of-stay prediction, readmission, deterioration	Tabular ML, EHR transformers, hybrid temporal models	SHAP, global interpretability methods, rule-based surrogates	AUROC, precision/recall, calibration, effect size estimates
Cross-Phase/Systems Level	Workflow optimization, robotic assistance, multimodal integration	Multimodal deep learning, graph neural networks (GNNs), video + EHR models	Saliency, SHAP for multimodal, prototype learning, concept-based explanations	Composite metrics; few studies report latency, human factors, or usability

**Table 11:** Limitations of LIME and SHAP in the surgical sphere.

	LIME	SHAP	Common Limitations
Approach	Local surrogate models for individual predictions	Shapley values from cooperative game theory	Both are post hoc explanation methods
Key Assumption	Relies on local linearity	Assumes feature independence	Not designed for temporal or streaming data
Sensitivity	Highly sensitive to data perturbation, leading to inconsistent explanations	May overlook spatial and temporal correlations in clinical data	Lack of robustness in dynamic surgical environments
Explainability Consistency	Varies across similar cases, undermining real-time clinical trust	More stable, but constrained by underlying assumptions	Can produce misleading or incomplete insights for clinicians
Streaming Data Support	Not natively supported	Not natively supported	Critical gap in intraoperative settings, like live endoscopy or sensor monitoring
Suitability for Surgery	Poor for real-time decision-making due to a lack of consistency	Limited in procedural workflows with complex and evolving inputs	Inadequate for high-frequency surgical data requiring rapid interpretability
Regulatory Alignment	Difficult to justify in auditable or legal contexts	Better aligned with auditability, but lacks full clinical interpretability	Neither fully complies with the transparency requirements for high-risk medical decisions

**Table 12:** Checklist mapping each study type to recognized frameworks, and indicating how XAI artifacts can be archived for audit under the EU AI Act/GDPR and device-quality systems.

Study Type	Frameworks	XAI Artifacts to Archive	Regulatory Hooks (EU AI Act/GDPR/Device Systems)
Model development	TRIPOD-AI, DECIDE Stage 0–1	Saliency maps, feature attribution logs, model cards, training data snapshots	GDPR minimization; AI Act documentation; ISO 14971 risk files
Retrospective validation	TRIPOD-AI, DECIDE Stage 2	Cohort-linked explanations, metadata, pseudonymized IDs	AI Act Art. 12 record-keeping; MDR Annex IV technical docs
Prospective feasibility	DECIDE Stage 3, IDEAL 2a/2b	Real-time explanations, clinician feedback, usability logs	IEC 62366 usability; AI Act transparency and audit trail
RCTs	SPIRIT-AI, CONSORT-AI, IDEAL 3	Full model+XAI version bundle; explanation reproducibility	MDR clinical evidence; AI Act transparency and data integrity
Post-market monitoring	IDEAL 4, AI Act Art. 61	Explanation-stream logs, drift detection, concept instability	AI Act post-market surveillance; ISO 14971 ongoing risk

**Table 13:** Frameworks and Regulatory References.

Frameworks	
TRIPOD-AI (Transparent Reporting of a multivariable prediction model for Individual Prognosis or Diagnosis—AI extension)	Provides reporting standards for developing and validating AI prediction models; ensures transparency, reproducibility, and clear disclosure of model performance and limitations.
DECIDE-AI (Developmental and Exploratory Clinical Investigation of Decision-support systems driven by AI)	Guides early-stage clinical evaluation of AI tools, focusing on human–AI interaction, workflow integration, and safety before definitive trials.
SPIRIT-AI (Standard Protocol Items: Recommendations for Interventional Trials—AI extension)	Sets standards for protocol design of clinical trials involving AI; ensures transparent, pre-registered trial methodology.
CONSORT-AI (Consolidated Standards of Reporting Trials—AI extension)	Provides detailed guidelines for reporting AI-enabled randomized controlled trials, including intervention description and AI model behavior.
IDEAL Framework (Idea, Development, Exploration, Assessment, Long-term Study)	Framework for evaluating surgical innovations; applicable to AI to structure translation from prototype to deployment and surveillance.
MISE/CLAIM/EPF (Medical Imaging and Signal Evaluation Frameworks)	Standards for evaluating imaging, sensor, and video-based AI models; emphasize dataset description, benchmarking, reproducibility, and methodological rigor.
PROBAST-AI (Prediction Model Risk of Bias Assessment Tool—AI Extension)	Tool for assessing risk of bias and applicability in AI prediction-model studies; identifies methodological weaknesses and overfitting.
EU AI Act	Regulates high-risk clinical AI systems; mandates documentation, transparency, human oversight, post-market monitoring, and auditable explanation logs.
GDPR (General Data Protection Regulation)	Governs personal-data handling; establishes requirements for pseudonymisation, data minimisation, storage of explanation artifacts, and privacy protection.
ISO 13485 (Medical Device Quality Management Systems)	Defines quality-management and documentation requirements for medical devices, including AI models and their XAI pipelines.
ISO 14971 (Risk Management for Medical Devices)	Requires systematic identification, analysis, and mitigation of risks—including risks introduced by misleading or unstable explanations.
IEC 62304 (Software Lifecycle Processes)	Specifies lifecycle management, version control, and maintenance requirements for medical device software, including AI models and XAI components.
IEC 62366 (Usability Engineering for Medical Devices)	Establishes usability engineering standards; ensures safe, effective clinician interaction with AI systems and interpretability interfaces.
MDR (EU Medical Device Regulation)	Regulates clinical evidence generation, technical documentation, and post-market surveillance for AI systems seeking CE marking.

**Table 14:** AI model validation: integrating causal-inference principles by a six-step framework: (1) define the causal question (intervention → outcome); (2) map the causal structure (using directed-acyclic graphs and domain expertise); (3) assess data readiness (temporal ordering, confounder measurement, reverse causality risks); (4) select causal-estimation methods (e.g., propensity scores, instruments, inverse-probability weighting), explicitly stating assumptions; (5) validate for transportability and robustness across settings; and (6) interpret results with careful language, distinguishing prediction from causation and signaling residual uncertainties.

Step	Question to Ask	Practical Actions in AI Model Validation
1. Clarify the causal question	What is the intervention/exposure, and what is the outcome? What is the causal effect we hope the model will support?	Define explicitly in the protocol; e.g., “If we do X (intervention), then Y (outcome) will change by Δ”. Avoid ambiguous phrasing like “predict Y from X” when the goal is to infer “X causes Y”.
2. Specify the causal structure	What variables are on the pathway, what are confounders, mediators, and colliders? Can we map a directed-acyclic graph (DAG) or a potential-outcome model?	Construct a DAG as part of model development; use domain knowledge (clinicians + data team) to list confounders or hidden variables. Use this to guide variable selection, adjustment strategy, and interpretation of model features.
3. Assess data adequacy for causal inference	Does the dataset support the assumptions needed for causal inference? What about: completeness of confounder measurement, absence of unmeasured confounding, timing of exposures/outcomes (temporal order), possibility of reverse causation?	Ensure that exposure precedes outcome in the data. Inspect for missing key confounders or proxies. Acknowledge and quantify potential unmeasured confounding (e.g., with sensitivity analyses). If retrospective data, consider issues of reverse causality.
4. Choose and apply appropriate causal estimation methods	Is a simple predictive model enough, or do we need causal-specific methods (e.g., propensity score matching/weighting, instrumental variables, inverse probability weighting)? What assumptions underlie each approach?	In AI model validation for clinical use: Use stratification or matching (or weighting) to balance covariates if the model aims to estimate the effect of an exposure. Evaluate whether an instrument is available if exposure is endogenous. Use techniques to mitigate bias (e.g., controlling for confounders, avoiding collider bias). Report the assumptions explicitly (e.g., no unmeasured confounding, stable unit-treatment value assumption).
5. Validate model results for transportability and robustness	Do the causal claims hold across populations, sites, and time periods? Does the AI model’s behavior change under shift (population, measurement, intervention)?	Perform external validation (other hospitals, different patient mix). Conduct sensitivity analyses; e.g., how much unobserved confounding would invalidate the conclusion? Report robustness to hidden bias. Check whether causal effect estimates (or model predictions) vary significantly under realistic shifts.
6. Interpret and communicate findings cautiously	Are we inadvertently making causal claims when only associations are supported? Are we clear about the limitations and remaining uncertainties?	In the manuscript: emphasize “This model predicts Y given X; it does not necessarily mean X causes Y unless assumptions hold.” Provide caveats about confounding, bias, and generalisability. Avoid words like “causes” unless the design supports it. Use language such as “may be associated with”, “in this dataset under these assumptions, an effect of X on Y is estimated as …”.

## Data Availability

No new data were created or analyzed in this study.

## References

[1] Saraiva M.M., Ribeiro T., Agudo B., Afonso J., Mendes F., Martins M., Cardoso P., Mota J., Almeida M.J., Costa A. (2025). Evaluating ChatGPT-4 for the Interpretation of Images from Several Diagnostic Techniques in Gastroenterology. J. Clin. Med..

[2] Dang F., Samarasena J.B. (2023). Generative Artificial Intelligence for Gastroenterology: Neither Friend nor Foe. Am. J. Gastroenterol..

[3] Hirosawa T., Kawamura R., Harada Y., Mizuta K., Tokumasu K., Kaji Y., Suzuki T., Shimizu T. (2023). ChatGPT-Generated Differential Diagnosis Lists for Complex Case-Derived Clinical Vignettes: Diagnostic Accuracy Evaluation. JMIR Med. Inform..

[4] Sonoda Y., Kurokawa R., Nakamura Y., Kanzawa J., Kurokawa M., Ohizumi Y., Gonoi W., Abe O. (2024). Diagnostic performances of GPT-4o, Claude 3 Opus, and Gemini 1.5 Pro in “Diagnosis Please” cases. Jpn. J. Radiol..

[5] Henson J.B., Glissen Brown J.R., Lee J.P., Patel A., Leiman D.A. (2023). Evaluation of the Potential Utility of an Artificial Intelligence Chatbot in Gastroesophageal Reflux Disease Management. Am. J. Gastroenterol..

[6] Gorelik Y., Ghersin I., Arraf T., Ben-Ishay O., Klein A., Khamaysi I. (2024). Using a customized GPT to provide guideline-based recommendations for management of pancreatic cystic lesions. Endosc. Int. Open.

[7] Javan R., Kim T., Mostaghni N. (2024). GPT-4 Vision: Multi-Modal Evolution of ChatGPT and Potential Role in Radiology. Cureus.

[8] Dehdab R., Brendlin A., Werner S., Almansour H., Gassenmaier S., Brendel J.M., Nikolaou K., Afat S. (2024). Evaluating ChatGPT-4V in chest CT diagnostics: A critical image interpretation assessment. Jpn. J. Radiol..

[9] Shifai N., van Doorn R., Malvehy J., Sangers T.E. (2024). Can ChatGPT vision diagnose melanoma? An exploratory diagnostic accuracy study. J. Am. Acad. Dermatol..

[10] Kokkinakis S., Kritsotakis E.I., Lasithiotakis K. (2023). Artificial Intelligence in Surgical Risk Prediction. J. Clin. Med..

[11] Chevalier O., Dubey G., Benkabbou A., Majbar M.A., Souadka A. (2025). Comprehensive overview of artificial intelligence in surgery: A systematic review and perspectives. Pflugers Arch..

[12] Li Y.Y., Wang J.J., Huang S.H., Kuo C.-L., Chen J.-Y., Liu C.-F., Chu C.-C. (2022). Implementation of a machine learning application in preoperative risk assessment for hip repair surgery. BMC Anesthesiol..

[13] Kinoshita M., Ueda D., Matsumoto T., Shinkawa H., Yamamoto A., Shiba M., Okada T., Tani N., Tanaka S., Kimura K. (2023). Deep Learning Model Based on Contrast-Enhanced Computed Tomography Imaging to Predict Postoperative Early Recurrence after the Curative Resection of a Solitary Hepatocellular Carcinoma. Cancers.

[14] Liu C.-Y., Cheng C.-Y., Yang S.-Y., Chai J.-W., Chen W.-H., Chang P.-Y. (2023). Mortality Evaluation and Life Expectancy Prediction of Patients with Hepatocellular Carcinoma with Data Mining. Healthcare.

[15] Kenig N., Monton Echeverria J., Muntaner Vives A. (2024). Artificial Intelligence in Surgery: A Systematic Review of Use and Validation. J. Clin. Med..

[16] Karamitros G., Thayer W.P., Lamaris G.A., Perdikis G., Lineaweaver W.C. (2025). Structural barriers and pathways to artificial intelligence integration in plastic surgery. J. Plast. Reconstr. Aesthet. Surg..

[17] Mienye I.D., Obaido G., Jere N., Mienye E., Aruleba K., Emmanuel I.D., Ogbuokiri B. (2024). A Survey of Explainable Artificial Intelligence in Healthcare: Concepts, Applications, and Challenges. Inform. Med. Unlocked.

[18] Zhang Y., Weng Y., Lund J. (2022). Applications of Explainable Artificial Intelligence in Diagnosis and Surgery. Diagnostics.

[19] Metta C., Beretta A., Pellungrini R., Rinzivillo S., Giannotti F. (2024). Towards Transparent Healthcare: Advancing Local Explanation Methods in Explainable Artificial Intelligence. Bioengineering.

[20] Brandenburg J.M., Müller-Stich B.P., Wagner M., van der Schaar M. (2025). Can surgeons trust AI? Perspectives on machine learning in surgery and the importance of eXplainable Artificial Intelligence (XAI). Langenbecks Arch. Surg..

[21] Amann J., Blasimme A., Vayena E., Frey D., Madai V.I. (2020). Explainability for artificial intelligence in healthcare: A multidisciplinary perspective. BMC Med. Inform. Decis. Mak..

[22] Hakkoum H., Idri A., Abnane I. (2024). Global and local interpretability techniques of supervised machine learning black box models for numerical medical data. Eng. Appl. Artif. Intell..

[23] Atzmueller M., Fürnkranz J., Kliegr T., Schmid U. (2024). Explainable and interpretable machine learning and data mining. Data Min. Knowl. Discov..

[24] Mohamed Y.A., Khoo B.E., Asaari M.S.M., Aziz M.E., Ghazali F.R. (2025). Decoding the black box: Explainable AI (XAI) for cancer diagnosis, prognosis, and treatment planning-A state-of-the art systematic review. Int. J. Med. Inform..

[25] Torda T., Ciardiello A., Gargiulo S., Grillo G., Scardapane S., Voena C., Giagu S. (2025). Influence based explainability of brain tumors segmentation in magnetic resonance imaging. Prog. Artif. Intell..

[26] Haupt M., Maurer M.H., Thomas R.P. (2025). Explainable Artificial Intelligence in Radiological Cardiovascular Imaging—A Systematic Review. Diagnostics.

[27] Song D., Yao J., Jiang Y., Shi S., Cui C., Wang L., Wang L., Wu H., Tian H., Ye X. (2023). A new xAI framework with feature explainability for tumors decision-making in Ultrasound data: Comparing with Grad-CAM. Comput. Methods Programs Biomed..

[28] Topol E. (2019). Deep Medicine: How Artificial Intelligence Can Make Healthcare Human Again.

[29] Mascarenhas M., Mendes F., Martins M., Ribeiro T., Afonso J., Cardoso P., Ferreira J., Fonseca J., Macedo G. (2025). Explainable AI in Digestive Healthcare and Gastrointestinal Endoscopy. J. Clin. Med..

[30] Plass M., Kargl M., Kiehl T.R., Regitnig P., Geißler C., Evans T., Zerbe N., Carvalho R., Holzinger A., Müller H. (2023). Explainability and causability in digital pathology. J. Pathol. Clin. Res..

[31] Salih A., Sengupta P.P. (2023). Explainable artificial intelligence and cardiac imaging. Circ. Cardiovasc. Imaging.

[32] Chanda T., Haggenmueller S., Bucher T.-C., Holland-Letz T., Kittler H., Tschandl P., Heppt M.V., Berking C., Utikal J.S., Schilling B. (2025). Dermatologist-like explainable AI enhances melanoma diagnosis accuracy: Eye-tracking study. Nat. Commun..

[33] Lundberg S.M., Nair B., Vavilala M.S., Horibe M., Eisses M.J., Adams T., Liston D.E., Low D.K.-W., Newman S.-F., Kim J. (2018). Explainable machine-learning predictions for the prevention of hypoxaemia during surgery. Nat. Biomed. Eng..

[34] Esteva A., Robicquet A., Ramsundar B., Kuleshov V., DePristo M., Chou K., Cui C., Corrado G., Thrun S., Dean J. (2019). A guide to deep learning in healthcare. Nat. Med..

[35] Holzinger A., Biemann C., Pattichis C.S., Kell D.B. (2017). What do we need to build explainable AI systems for the medical domain?. arXiv.

[36] Tjoa E., Guan C. (2020). A Survey on Explainable Artificial Intelligence (XAI): Towards Medical XAI. IEEE Trans. Neural Netw. Learn. Syst..

[37] Gerke S., Minssen T., Cohen G. (2020). Ethical and legal challenges of artificial intelligence-driven healthcare. Artificial Intelligence in Healthcare.

[38] Price W.N.I.I., Gerke S., Cohen I.G. (2019). Potential liability for physicians using artificial intelligence. JAMA.

[39] Yu K.H., Kohane I.S. (2019). Framing the challenges of artificial intelligence in medicine. BMJ Qual. Saf..

[40] Ross C., Swetlitz I. (2018). IBM’s Watson supercomputer recommended “unsafe and incorrect” cancer treatments, internal documents show. Stat News..

[41] European Commission (2021). Proposal for a Regulation Laying Down Harmonised Rules on Artificial Intelligence (AI Act). https://digital-strategy.ec.europa.eu/en/library/proposal-regulation-laying-down-harmonised-rules-artificial-intelligence.

[42] Challen R., Denny J., Pitt M., Gompels L., Edwards T., Tsaneva-Atanasova K. (2019). Artificial intelligence, bias and clinical safety. BMJ Qual. Saf..

[43] Morley J., Floridi L. (2020). An ethically mindful approach to AI for health care. Lancet Digit Health.

[44] Holzinger A., Langs G., Denk H., Zatloukal K., Müller H. (2019). Causability and explainability of AI in medicine. Wiley Interdiscip. Rev. Data Min. Knowl. Discov..

[45] Lundberg S.M., Lee S.I. (2017). A unified approach to interpreting model predictions. Adv. Neural Inf. Process. Syst..

[46] Wachter S., Mittelstadt B., Russell C. (2018). Counterfactual explanations without opening the black box: Automated decisions and the GDPR. Harv. J. Law Technol..

[47] Coiera E. (2017). The forgetting health system. Learn Health Syst..

[48] Yu K.H., Beam A.L., Kohane I.S. (2018). Artificial intelligence in healthcare. Nat. Biomed. Eng..

[49] London A.J. (2019). Artificial intelligence and black-box medical decisions: Accuracy versus explainability. Hastings Cent. Rep..

[50] Ghassemi M., Oakden-Rayner L., Beam A.L. (2021). The false hope of current approaches to explainable artificial intelligence in health care. Lancet Digit Health.

[51] Shortliffe E.H., Sepúlveda M.J. (2018). Clinical decision support in the era of artificial intelligence. JAMA.

[52] Longoni C., Bonezzi A., Morewedge C.K. (2019). Resistance to medical artificial intelligence. J. Consum. Res..

[53] Tonekaboni S., Joshi S., McCradden M.D., Goldenberg A. (2019). What clinicians want: Contextualizing explainable machine learning for clinical end use. arXiv.

[54] Lundberg S.M., Erion G., Lee S.I. (2018). Consistent individualized feature attribution for tree ensembles. arXiv.

[55] Vayena E., Blasimme A., Cohen I.G. (2018). Machine learning in medicine: Addressing ethical challenges. PLoS Med..

[56] Obermeyer Z., Powers B., Vogeli C., Mullainathan S. (2019). Dissecting racial bias in an algorithm used to manage the health of populations. Science.

[57] Haider A.H., Scott V.K., Rehman K.A., Velopulos C., Bentley J.M., Cornwell E.E., Al-Refaie W. (2013). Racial disparities in surgical care and outcomes in the United States: A comprehensive review. J. Am. Coll. Surg..

[58] Rajkomar A., Hardt M., Howell M.D., Corrado G., Chin M.H. (2018). Ensuring fairness in machine learning to advance health equity. Ann. Intern. Med..

[59] Chen I.Y., Joshi S., Ghassemi M. (2020). Treating health disparities with artificial intelligence. Nat. Med..

[60] Seyyed-Kalantari L., Zhang H., McDermott M., Chen I.Y., Ghassemi M. (2021). Underdiagnosis bias of artificial intelligence algorithms applied to chest radiographs in under-served patient populations. Nat. Med..

[61] Wiens J., Saria S., Sendak M., Ghassemi M., Liu V.X., Doshi-Velez F., Jung K., Heller K., Kale D., Saeed M. (2019). Do no harm: A roadmap for responsible machine learning for health care. Nat. Med..

[62] Mitchell M., Wu S., Zaldivar A., Barnes P., Vasserman L., Hutchinson B., Spitzer E., Raji I.D., Gebru T. Model cards for model reporting. Proceedings of the Conference on Fairness, Accountability, and Transparency (FAT).

[63] Wachter S., Mittelstadt B., Russell C. (2021). Why fairness cannot be automated: Bridging the gap between EU non-discrimination law and AI. Comput. Law Secur. Rev..

[64] Goulas S., Karamitros G. (2024). How to harness the power of web scraping for medical and surgical research: An application in estimating international collaboration. World J. Surg..

[65] Dzindolet M.T., Peterson S.A., Pomranky R.A., Pierce L.G., Beck H.P. (2003). The role of trust in automation reliance. Int. J. Hum. Comput. Stud..

[66] Elish M.C. (2019). Moral crumple zones: Cautionary tales in human-robot interaction. Engag. Sci. Technol. Soc..

[67] Sendak M.P., D’Arcy J., Kashyap S., Gao M., Nichols M., Corey K., Ratliff W. (2020). A path for translation of machine learning products into healthcare delivery. EMJ Innov..

[68] Amann J., Vetter D., Blomberg S.N., Christensen H.C., Coffee M., Gerke S., Gilbert T.K., Hagendorff T., Holm S., Livne M. (2022). To explain or not to ex-plain?—Artificial Intelligence Explainability in clinical decision support systems. PLoS Digit. Health.

[69] Mahajan A., Esper S., Oo T.H., McKibben J., Garver M., Artman J., Klahre C., Ryan J., Sadhasivam S., Holder-Murray J. (2023). Development and Validation of a Machine Learning Model to Identify Patients Before Surgery at High Risk for Postoperative Adverse Events. JAMA Netw. Open.

[70] Byrd IV T., Tignanelli C. (2024). Artificial intelligence in surgery—A narrative review. J. Med. Artif. Intell..

[71] Komorowski M., Celi L.A., Badawi O., Gordon A.C., Faisal A.A. (2018). The artificial intelligence clinician learns optimal treatment strategies for sepsis in intensive care. Nat. Med..

[72] Ribeiro M.T., Singh S., Guestrin C. “Why should I trust you?”: Explaining the predictions of any classifier. Proceedings of the 22nd ACM SIGKDD International Conference on Knowledge Discovery and Data Mining.

[73] Elwyn G., Frosch D., Thomson R., Joseph-Williams N., Lloyd A., Kinnersley P., Cording E., Tomson D., Dodd C., Rollnick S. (2012). Shared decision making: A model for clinical practice. J. Gen. Intern. Med..

[74] Selvaraju R.R., Cogswell M., Das A., Vedantam R., Parikh D., Batra D. Grad-CAM: Visual explanations from deep networks via gradient-based localization. Proceedings of the 2017 IEEE International Conference on Computer Vision (ICCV).

[75] Cizmic A., Mitra A.T., Preukschas A.A., Kemper M., Melling N.T., Mann O., Markar S., Hackert T., Nickel F. (2025). Artificial intelligence for intraoperative video analysis in robotic-assisted esophagectomy. Surg. Endosc..

[76] Hashimoto D.A., Rosman G., Rus D., Meireles O.R. (2018). Artificial intelligence in surgery: Promises and perils. Ann. Surg..

[77] Leszczyńska A., Obuchowicz R., Strzelecki M., Seweryn M. (2025). The Integration of Artificial Intelligence into Robotic Cancer Surgery: A Systematic Review. J. Clin. Med..

[78] Vasey B., Lippert K.A.N., Khan D.Z., Ibrahim M., Koh C.H., Layard Horsfall H., Lee K.S., Williams S., Marcus H.J., McCulloch P. (2023). Intraoperative Applications of Artificial Intelligence in Robotic Surgery: A Scoping Review of Current Development Stages and Levels of Autonomy. Ann. Surg..

[79] Riva-Cambrin H.A., Singh R., Lama S., Sutherland G.R. (2025). Liquid white box model as an explainable AI for surgery. Npj Digit. Med..

[80] Hernandez M.C., Chen C., Nguyen A., Choong K., Carlin C., Nelson R.A., Rossi L.A., Seth N., McNeese K., Yuh B. (2024). Explainable Machine Learning Model to Preoperatively Predict Postoperative Complications in Inpatients With Cancer Undergoing Major Operations. JCO Clin. Cancer Inform..

[81] Lopez-Lopez V., Rihuete-Carpio R., Herrero-Sánchez A., Gavara C.G., Goh B.K., Koh Y.X., Paul S.J., Hilal M.A., Mishima K., Krürger J.A.P. (2024). Explainable artificial intelligence prediction-based model in laparoscopic liver surgery for segments 7 and 8. Surg. Endosc..

[82] Wang S., Liu J., Wu Z., Liang P., Luo Z., Kong J., Huang J., Cheng M., Zhang B., Wang Y. (2025). Prediction model for postoperative pulmonary complications after thoracoscopic surgery with machine learning algorithms and SHapley Additive exPlanations (SHAP). J. Thorac. Dis..

[83] Fransvea P., Fransvea G., Liuzzi P., Sganga G., Mannini A., Costa G. (2022). Study and validation of an explainable machine learning–based mortality prediction following emergency surgery in the elderly: A prospective observational study (FRAILESEL). Int. J. Surg..

[84] Deng H., Liu Y., Liang Z., Veerapong J., Fournier K.F., Johnston F.M., Dineen S.P., Powers B.D., Hendrix R., Lambert L.A. (2022). Development and Validation of an Explainable Machine Learning Model for Major Complications After Cytoreductive Surgery. JAMA Netw. Open.

[85] Zeng X., Hu Y., Shu L., Li J., Duan H., Shu Q., Li H. (2021). Explainable machine-learning predictions for complications after pediatric congenital heart surgery. Sci. Rep..

[86] Arabian H., Alshirbaji T.A., Jalal N.A., Krueger-Ziolek S., Moeller K. (2023). P-CSEM: An Attention Module for Improved Laparoscopic Surgical Tool Detection. Sensors.

[87] Shinozuka K., Toruida S., Fujinaga A., Nakanuma H., Kawamura M., Matsunobu Y., Tanaka Y., Kamiyama T., Ebe K., Endo Y. (2022). Artificial intelligence software available for medical devices: Surgical phase recognition in laparoscopic cholecystectomy. Surg. Endosc..

[88] Hashimoto D.A., Rosman G., Witkowski E.R. (2019). Computer Vision Analysis of Intraoperative Video: Automated Recognition of Operative Steps in Laparoscopic Sleeve Gastrectomy. Ann. Surg..

[89] Madani A., Namazi B., Altieri M.S., Hashimoto D.A., Rivera A.M., Pucher P.H., Navarrete-Welton A., Sankaranarayanan G., Brunt L.M., Okrainec A. (2022). Artificial Intelligence for Intraoperative Guidance: Using Semantic Segmentation to identify surgical anatomy during Laparoscopic Cholecystectomy. Ann. Surg..

[90] Zia A., Essa I. (2018). Automated surgical skill assessment in RMIS training. Int. J. Comput. Assist. Radiol. Surg..

[91] Funke I., Mees S.T., Weitz J., Speidel S. (2019). Video-based surgical skill assessment using 3D convolutional neural networks. Int. J. Comput. Assist. Radiol. Surg..

[92] Lam K., Chen J., Wang Z., Iqbal F.M., Darzi A., Lo B., Purkayastha S., Kinross J.M. (2022). MAchine Learning for technical skill assessment in surgery: A systematic review. npj Digit. Med..

[93] Mascagni P., Alapatt D., Sestini L. (2022). Computer vision in surgery: From potential to clinical value. npj Digi. Med..

[94] Sarker S.K., Chang A., Vincent C., Darzi S.A. (2006). Development of assessing generic and specific technical skills in laparoscopic surgery. Am. J. Surg..

[95] Rudin C. (2019). Stop explaining black box machine learning models for high stakes decisions and use interpretable models instead. Nat. Mach. Intell..

[96] Slack D., Hilgard S., Jia E., Singh S., Lakkaraju H. Fooling LIME and SHAP: Adversarial attacks on post hoc explanation methods. Proceedings of the AAAI/ACM Conference on AI, Ethics, and Society.

[97] Sendak M.P., Ratliff W., Sarro D., Alderton E., Futoma J., Gao M., Nichols M., Revoir M., Yashar F., Miller C. (2020). Real-world Integration of a sepsis Deep Learning Technology Into Routine Clinical Care: Implementation Study. JMIR MED Inform..

[98] Topol E.J. (2019). High-performance medicine: The convergence of human and artificial intelligence. Nat. Med..

[99] Ratwani R.M., Reider J., Singh H. (2019). A decade of health information technology usability challenges and the path forward. JAMA.

[100] Holzinger A., Kieseberg P., Weippl E., Tjoa A.M. (2018). Current advances, trends and challenges of machine learning and knowledge extraction: From machine learning to explainable AI. Machine Learning and Knowledge Extraction.

[101] Padoy N. (2019). Machine and deep learning for workflow recognition during surgery. Minim. Invasive Ther. Allied Technol..

[102] U.S. Food & Drug Administration (2021). Artificial Intelligence/Machine Learning (AI/ML)-Based Software as a Medical Device (SaMD): Action Plan. https://www.fda.gov/media/145022/download.

[103] (2021). FDA Discussion Paper: Proposed Regulatory Framework for Modifications to AI/ML-Based Software as a Medical Device. https://www.fda.gov/files/medical%20devices/published/US-FDA-Artificial-Intelligence-and-Machine-Learning-Discussion-Paper.pdf.

[104] Laux J., Watcher S., Mittelstadt B. (2024). Trustworthy artificial intelligence and the European Union AI Act: On the conflation of trustworthiness and the acceptability of risk. Regul. Gov..

[105] Rad A.A., Vardanyan R., Athanasiou T., Maessen J., Nia P.S. (2025). The Ethical Considerations of integrating artificial intelligence into surgery: A review. Interdiscip. Cardiovasc. Thorac. Surg..

[106] Karamitros G., Grant M.P., Lamaris G.A. (2025). Associations in Medical Research Can Be Misleading: A Clinician’s Guide to Causal Inference. J. Surg. Res..

[107] Shen J., Xue B., Kannampallil T., Lu C., Abraham J. (2024). A Novel Generative Multi-Task Representation Learning Approach for Predicting Postoperative Complications in Cardiac Surgery Patients. arXiv.

[108] Shen J., Xue B., Kannampallil T., Lu C., Abraham J. (2025). A novel generative multi-task representation learning approach for predicting postoperative complications in cardiac surgery patients. J. Am. Med. Inform. Assoc..

[109] Vasey B., Nagendran M., Campbell B., Clifton D., Collins G.S., Denaxas S., Denniston A.K., Faes L., Geerts B., Ibrahim M. (2022). Reporting guideline for the early-stage clinical evaluation of decision support systems driven by artificial intelligence: DECIDE-AI. BMJ.

